# When air gets heavy: joint PM_2.5_ and ambient humidity exposure, frailty, and stroke

**DOI:** 10.3389/fpubh.2026.1883147

**Published:** 2026-09-09

**Authors:** Min Sun, Jianbin Lin, Yang Cao, Xieli Guo, Jiateng Zeng, Pengwei Hou

**Affiliations:** 1 Department of Neurosurgery, Jinjiang Municipal Hospital (Shanghai Sixth People's Hospital Fujian), Jinjiang, Fujian, China; 2 Department of Neurosurgery, The Fifth Hospital of Xiamen, Xiamen, Fujian, China; 3 Department of Neurosurgery, Neurosurgery Research Institute, The First Affiliated Hospital, Fujian Medical University, Fuzhou, Fujian, China

**Keywords:** ambient humidity, CHARLS, frailty, mediation analysis, PM_2.5_, stroke

## Abstract

**Background:**

PM_2.5_ exposure has been associated with stroke, but evidence regarding ambient humidity, joint environmental exposure, and the mediating role of frailty remains limited.

**Methods:**

We integrated data from the China Health and Retirement Longitudinal Study (CHARLS; *n* = 17,197) and a single-center hospital-based sample (*n* = 782). Annual average PM2.5 and relative humidity in the year before baseline or index date were assigned by residential location and dichotomized using database-specific medians. Joint exposure was categorized into four groups. Incident stroke in CHARLS was analyzed using Cox models, and stroke status in the hospital sample using logistic regression. Counterfactual mediation analysis evaluated frailty index as a mediator. Model-based mediation analyses evaluated frailty as a potential mediator, with the hospital-based analysis treated as exploratory.

**Results:**

In fully adjusted models, high PM_2.5_ was associated with higher stroke risk in CHARLS (HR 1.184, 95% CI 1.083–1.295) and higher stroke odds in the hospital sample (OR 1.846, 95% CI 1.247–2.733). High humidity was also associated with stroke in CHARLS (HR 1.112, 95% CI 1.021–1.212) and the hospital sample (OR 1.928, 95% CI 1.301–2.859). Participants exposed to both high PM2.5 and high humidity had the highest estimates in CHARLS (HR 1.361, 95% CI 1.171–1.582) and the hospital sample (OR 2.431, 95% CI 1.390–4.251). Frailty mediated 18.1 and 22.3% of PM_2.5_- and humidity-related associations in CHARLS, and 15.8 and 17.0% in the hospital sample.

**Conclusion:**

Higher PM_2.5_ and ambient humidity were independently and jointly associated with greater stroke risk. Frailty partly mediated these associations, highlighting a clinically relevant pathway linking environmental exposure to stroke.

## 1 Introduction

Stroke remains one of the leading causes of death and disability worldwide and continues to impose a particularly heavy burden on ageing populations. A recent global fact sheet estimated that in 2021 there were 11.9 million incident strokes, 93.8 million prevalent strokes, and 7.3 million deaths from stroke worldwide, and that environmental risks accounted for 37.0% of all strokes (1). The latest Global Burden of Disease analysis likewise showed that stroke was the third leading level-3 cause of death globally in 2021, with much of the burden concentrated in lower-income settings (2). In China, where population ageing, environmental exposure, and stroke burden intersect at scale, these observations make the environmental determinants of stroke especially relevant.

Among environmental exposures, ambient fine particulate matter (PM_2.5_) has been linked most consistently to stroke. A systematic review and meta-analysis of 16 cohort studies including more than 2.2 million participants and over 49,149 stroke endpoints reported a pooled hazard ratio (HR) of 1.11 (95% CI 1.05–1.17) per 5 μg/m^3^ increase in PM_2.5_ for stroke incidence (3). In a large Chinese prospective cohort from the China-PAR project that included 117,575 adults without stroke at baseline, 3,540 incident strokes were identified during follow-up, and each 10 μg/m^3^ increase in PM_2.5_ exposure was associated with an HR of 1.13 (95% CI 1.09–1.17) for incident stroke (4). A pooled European analysis of 137,148 adults from six cohorts, with 6,950 incident strokes over a median follow-up of 17.2 years, also found a positive association at relatively low exposure levels (HR 1.10, 95% CI 1.01–1.21 per 5 μg/m^3^) (5). Less is known, however, about how PM_2.5_ may relate to stroke when considered together with persistently humid ambient conditions.

By contrast, the evidence on humidity and stroke is far more mixed. A systematic review and meta-analysis of observational studies reported pooled odds ratios (ORs) of 1.00 (95% CI 1.00–1.01) for ischemic stroke and 1.00 (95% CI 0.99–1.01) for intracerebral hemorrhage per 1% increase in relative humidity, with no clear overall evidence for an association (6). More recently, a time-stratified case-crossover study based on the Israeli National Stroke Registry, including 22,269 individuals with a first stroke and 8,728 with a first transient ischemic attack, found that 90% versus 70% relative humidity was associated with a higher stroke risk at lag 2 (OR 1.09, 95% CI 1.06–1.12) (7). That same study also reported an approximately 40% higher risk of stroke on the event day for a heat index of 100 °F versus 80 °F (OR 1.39, 95% CI 1.32–1.47) (7). Most of this literature, however, has focused on short-term meteorological conditions around the event date rather than annual average humidity in the residential environment.

Ambient relative humidity was nevertheless selected *a priori* as a primary exposure because it is a physiologically relevant component of the thermal environment rather than merely a descriptive meteorological variable. Particularly under warm conditions, higher relative humidity reduces the water-vapor pressure gradient between the skin and the surrounding air, thereby limiting evaporative heat loss and reducing the cooling efficiency of sweating. Maintaining thermal balance under humid conditions may consequently require greater cutaneous vasodilation and cardiovascular effort, while sustained sweating with inefficient evaporation may contribute to fluid and electrolyte imbalance, plasma-volume contraction, and blood-pressure instability (8–10). These physiological demands may be especially relevant to middle-aged and older adults, in whom sweating capacity, cutaneous vasodilation, thirst perception, and cardiovascular reserve are often diminished (10). Systematic evidence has linked heat exposure to increased cardiovascular morbidity and mortality, including stroke, and a recent stroke-focused review has further emphasized the clinical relevance of meteorological and climate-related stressors (8, 9). Nevertheless, relative humidity is inherently dependent on ambient temperature, and its biological effects are likely to vary according to the surrounding thermal context. We therefore considered annual average relative humidity as an indicator of the longer-term residential climatic moisture environment, rather than as a direct measure of acute humid heat or an established causal risk factor.

Frailty may offer a clinically meaningful bridge between environmental stressors and stroke. As a state of multisystem vulnerability characterized by diminished physiological reserve, frailty has direct relevance to cerebrovascular susceptibility and recovery. A recent systematic review and meta-analysis including 11 studies and 1,660,328 participants showed that pre-stroke frailty was associated with a significantly higher risk of stroke (pooled HR 1.72, 95% CI 1.46–2.02) (11). At the same time, frailty itself appears to be environmentally patterned: in a nationwide prospective cohort from the Chinese Longitudinal Healthy Longevity Survey including 25,047 adults aged 65 years or older, 5,733 incident frailty cases occurred during 107,814.8 person-years of follow-up, and each 10 μg/m^3^ increase in PM_2.5_ was associated with a 5% higher risk of frailty (HR 1.05, 95% CI 1.03–1.07) (12). A recent UK Biobank analysis of 444,094 participants further suggested that frailty partly mediated the association of PM_2.5_ with later depression and anxiety, indicating that frailty may function not only as a vulnerability marker but also as a pathway variable in air-pollution epidemiology (13). Direct epidemiological evidence linking long-term ambient humidity to frailty remains sparse. However, when elevated humidity repeatedly co-occurs with warm conditions, impaired evaporative cooling and the associated cardiovascular and fluid-regulatory demands described above may act as recurrent physiological stressors. In individuals with limited physiological reserve, repeated stress accompanied by incomplete recovery may contribute to exhaustion, functional decline, and the progressive accumulation of health deficits that characterizes frailty (10, 14). This broader thermal-stress hypothesis is supported indirectly by a national longitudinal study in which heatwave exposure was associated with a higher incidence of frailty (15). Because that study evaluated heatwaves rather than relative humidity itself, we regard the proposed long-term humidity–frailty pathway as biologically plausible but not yet established.

Despite this growing literature, several gaps remain. The evidence for PM_2.5_ and stroke is now substantial, but corresponding evidence for ambient humidity is limited and far less consistent, and studies that examine their joint associations are scarce. In addition, although frailty is linked to both environmental exposure and stroke risk, direct evidence for a long-term humidity–frailty association remains limited, and frailty has rarely been evaluated as a potential mediator in this context. Accordingly, we hypothesized that higher PM_2.5_ exposure and higher annual average relative humidity would each be associated with less favorable stroke outcomes, that their joint presence would show the strongest association with stroke, and that frailty would account for part of these associations. Using data from the China Health and Retirement Longitudinal Study (CHARLS) and a single-center hospital-based study, we therefore aimed to investigate the separate and joint associations of ambient PM_2.5_ exposure and ambient relative humidity with stroke, and to evaluate frailty as a potential mediator of these associations.

## 2 Methodology

### 2.1 Study design and data sources

This study integrated two data sources: the China Health and Retirement Longitudinal Study (CHARLS) and a single-center hospital-based clinical dataset from Jinjiang Municipal Hospital (Shanghai Sixth People’s Hospital Fujian). The study aimed to examine the separate and joint associations of PM_2.5_ exposure and ambient relative humidity with stroke and to evaluate frailty as a potential mediator across a population-based prospective cohort and a clinical cross-sectional sample.

CHARLS is a nationally representative longitudinal survey of Chinese adults aged 45 years and older and their spouses or partners (16). It was established to characterize the health, socioeconomic conditions, and demographic features of middle-aged and older populations living in communities across China. The baseline survey was conducted in 2011 using a multistage, probability-based, stratified cluster sampling design that covered 150 county-level units across 28 provincial-level regions. Follow-up surveys were completed in 2013, 2015, 2018, and 2020, with repeated collection of demographic, behavioral, and health-related information. The CHARLS component of the present study was designed as a prospective cohort study for incident stroke. The hospital component was designed as a single-center hospital-based cross-sectional study comparing patients with clinically diagnosed stroke and hospitalized non-stroke controls.

### 2.2 Participants

For the population-based cohort, 25,823 potentially eligible participants were screened. We excluded 3,567 participants with missing stroke information, 815 with stroke already present at baseline, 1,931 without valid follow-up information, 523 with missing frailty information, and 1,790 with missing covariates. No participants were excluded because of missing environmental exposure assignment. The final analytic sample for the cohort component comprised 17,197 participants. Follow-up accrued from the baseline interview date until the earliest of incident stroke, death, loss to follow-up, or the administrative end of follow-up.

For the single-center hospital study, 1,148 hospitalized adults were consecutively screened between June 2023 and August 2025. A total of 66 participants with incomplete clinical documentation, 159 with unavailable or invalid residential information for environmental exposure linkage, 52 with uncertain cerebrovascular diagnosis or transient neurological presentation without definitive stroke classification, 44 with missing frailty data, and 45 with missing covariates were excluded. The final analytic hospital sample therefore included 782 participants, comprising 148 patients with stroke and 634 hospitalized non-stroke controls.

**Figure 1:**
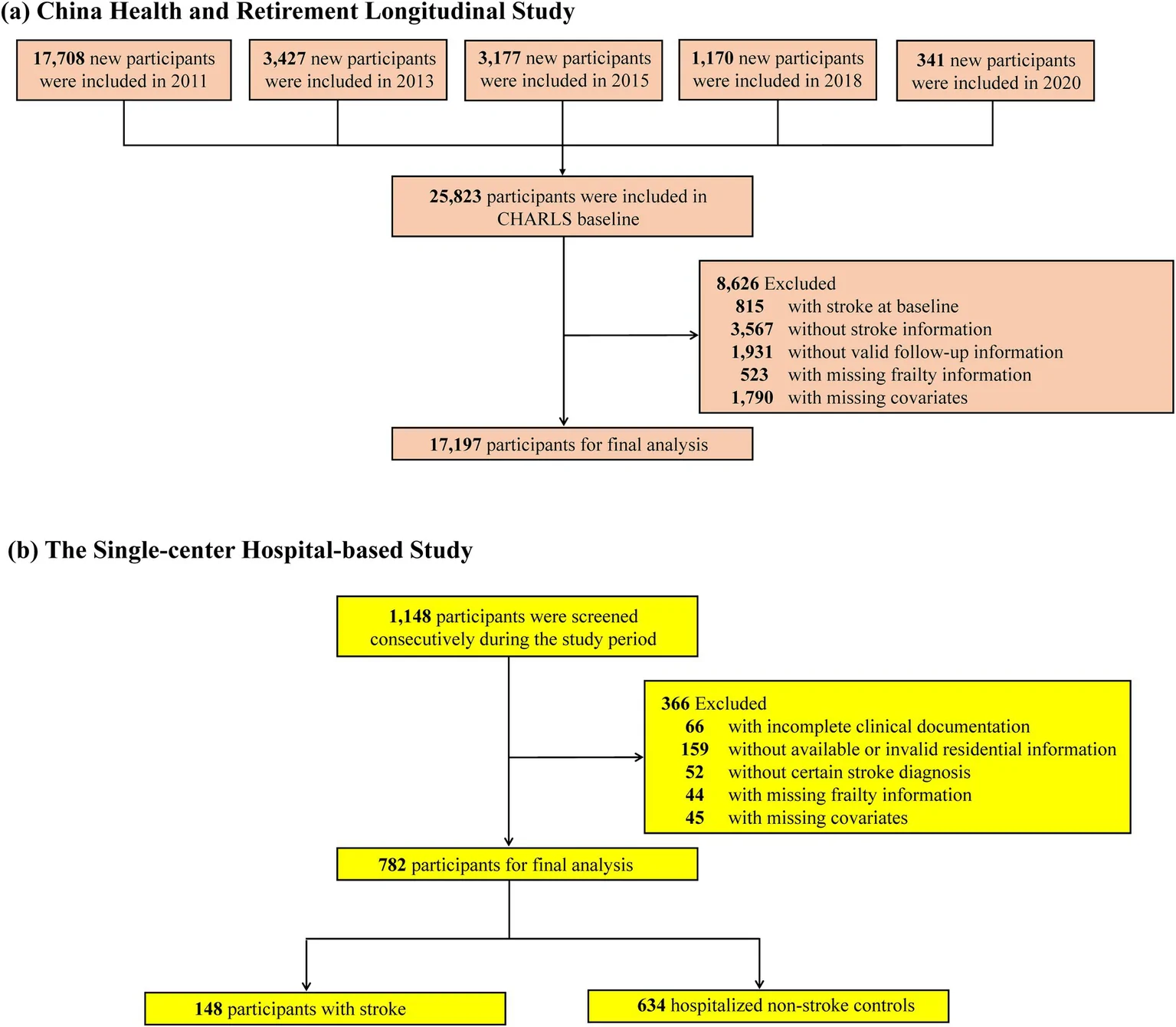
Flowchart of participant selection in CHARLS and the single-center hospital-based study. Panel **(a)** shows the derivation of the analytic sample for the China Health and Retirement Longitudinal Study (CHARLS). Panel **(b)** shows the derivation of the analytic sample for the single-center hospital-based study.

See Figure 1 for the selection process of CHARLS and the single-center hospital-based study.

### 2.3 Exposure assessment: PM_2.5_ and ambient relative humidity exposures

The primary environmental exposures were ambient PM_2.5_ and ambient relative humidity. PM_2.5_ was selected because fine particulate matter has been consistently linked to cardiovascular, inflammatory, and aging-related processes that are relevant to both frailty and stroke. Annual PM_2.5_ concentrations were obtained from the China High Air Pollutants (CHAP) dataset,^1^ which provides high-resolution gridded air-pollution estimates across China and has been widely used in environmental epidemiology studies (17). CHAP offers year-by-year estimates of PM_2.5_ across China at a 1-km spatial resolution, and the source product has demonstrated high predictive performance in cross-validation analyses.

In the cohort component, each participant was assigned the annual average PM_2.5_ concentration for the calendar year immediately preceding the baseline interview year according to geocoded residential community information. Thus, participants entering the study in 2011 were assigned the annual average PM_2.5_ concentration for 2010 (18). In the single-center hospital study, the same lag structure was applied by linking the residential city or address recorded at the index hospitalization to the same gridded air-pollution surface and assigning the annual average PM_2.5_ concentration for the year immediately preceding the index date.

Ambient relative humidity and ambient temperature were derived from the ERA5 single-level dataset at a spatial resolution of 0.25° × 0.25°, corresponding to a nominal horizontal resolution of approximately 31 km (19). Hourly 2-m air temperature and 2-m dew-point temperature were extracted, and relative humidity was calculated using a standard saturation-vapor-pressure equation. Hourly values were aggregated to daily means and subsequently to annual means for the calendar year immediately preceding baseline or the hospital index date. In CHARLS, exposure estimates were linked to the geocoded centroid of each participant’s residential community. In the hospital-based study, residential addresses were geocoded to the finest available location. Complete or street-level addresses were available for 681 participants (87.1%), whereas the centroid of the recorded county or district was used for 101 participants (12.9%). ERA5 values were assigned by bilinear interpolation from the surrounding grid cells. PM_2.5_ was assigned from the 1-km CHAP surface using the same residential coordinates and one-year lagged exposure window.

Annual mean relative humidity was prespecified as the primary humidity metric because the study was designed to characterize the preceding-year residential climatic moisture environment rather than short-term meteorological triggers around stroke onset. Averaging daily relative humidity over a full calendar year provides a summary of the year-round regional humidity context and limits the influence of isolated day-to-day weather fluctuations. The use of the same one-year lagged window for relative humidity and PM_2.5_ also ensured temporal alignment between the two exposures in the separate and joint exposure analyses. We selected this annual metric rather than an event-day or short-lag measure because the primary study question concerned longer-term residential environmental conditions and baseline frailty, rather than the acute triggering of stroke by individual weather events. Annual mean relative humidity should therefore be interpreted as an indicator of the broader residential moisture climate and not as a measure of acute humid heat, seasonal humidity extremes, or indoor humidity. Because relative humidity is temperature-dependent, its physiological relevance also depends on the surrounding thermal context.

For the primary analyses, annual mean PM_2.5_ and annual mean relative humidity were each dichotomized using the database-specific median. Values below the median were classified as low and values at or above the median were classified as high. A four-category joint exposure variable was then constructed by cross-classifying PM_2.5_ and relative humidity as low PM_2.5_–low humidity, high PM_2.5_–low humidity, low PM_2.5_–high humidity, and high PM_2.5_–high humidity. In all regression models, low PM_2.5_–low humidity was used as the reference category. In secondary analyses, PM_2.5_ and relative humidity were additionally modeled as continuous variables.

### 2.4 Outcome ascertainment

In the cohort component, stroke was identified using the physician-diagnosis item asking whether a doctor had ever told the participant that he or she had had a stroke. Participants reporting stroke at baseline were treated as having prevalent stroke and were excluded. Among those free of stroke at study entry, incident stroke was defined as the first follow-up wave at which physician-diagnosed stroke was newly reported. Because the exact date of stroke onset was not collected in CHARLS, incident stroke was known to have occurred within the interval between the last stroke-free interview and the first interview at which stroke was reported. For the primary analysis in this study, the date of the first follow-up interview reporting stroke was used as the event-time marker. The resulting time-to-event measure should therefore be interpreted as time to the first ascertainment of incident stroke rather than the exact biological onset time. Stroke subtype information was not available in CHARLS because incident stroke was ascertained from a general physician-diagnosis item.

In the single-center hospital-based study, stroke status was determined from the clinician-documented discharge diagnosis and was independently verified using the inpatient neurological assessment and brain computed tomography or magnetic resonance imaging. Stroke cases included acute ischemic stroke, intracerebral hemorrhage, and subarachnoid hemorrhage. Transient ischemic attacks, silent or chronic infarcts without an acute clinical syndrome, traumatic intracranial hemorrhage, and presentations without a definitive cerebrovascular diagnosis were excluded. Hospitalized non-stroke controls were consecutively enrolled during the same study period from general internal medicine, geriatrics, cardiology, endocrinology, respiratory medicine, gastroenterology, and orthopedics. Controls had no current or previous documented stroke, no admission for an acute cerebrovascular syndrome, and no clinical or imaging evidence of an acute stroke at the index hospitalization. Control selection was completed without knowledge of the assigned environmental exposure values.

### 2.5 Mediator: frailty

Frailty was assessed at baseline or index assessment and treated as the prespecified mediator. In both data sources, frailty was operationalized using a deficit-accumulation frailty index constructed from 40 health deficits (20, 21). These deficits covered multiple domains of aging-related vulnerability, including activities of daily living and instrumental activities of daily living, physical functioning, chronic diseases, psychological status, self-rated health, and related indicators of physiological reserve. Each deficit was coded on a scale from 0 to 1 according to standard deficit-accumulation principles, and the overall frailty index was calculated as the sum of deficits divided by the number of deficits assessed. The resulting index ranged from 0 to 1, with higher values indicating greater frailty burden. In the single-center hospital-based study, frailty deficits were coded to reflect the participant’s usual pre-stroke or pre-admission health status on the basis of the admission record, past medical history, pre-admission functional information, and patient or family report; acute stroke-related deficits and in-hospital complications were excluded. Detailed components and coding rules used to construct the frailty index are provided in Appendix 1.

For descriptive reporting, frailty was additionally classified as non-frail and frail using the conventional threshold of frailty index ≥0.25 (20). For mediation analyses, the continuous frailty index was used as the primary mediator.

The frailty index and the comorbidity index served distinct analytical roles. Frailty was modeled as the prespecified mediator in separate mediation analyses, whereas the comorbidity index was used only as an additional adjustment variable in a sensitivity analysis. The two indices were not entered simultaneously in the same exposure–stroke model; therefore, overlapping disease indicators were not double-counted or given additional statistical weight.

### 2.6 Covariates

The covariates included age, sex, marital status, residence, smoking status, alcohol drinking status, income group, sleep duration group, number of living children, and employment status. Age was modeled as a continuous variable. Sex was coded as female or male. Marital status was classified as married versus other or unknown. Residence was classified as rural or urban. Income was categorized as below median and at or above median based on the study-specific income distribution; in CHARLS, income was dichotomized at the non-zero median because zero income values were common. The corresponding cut-off values were CNY 4,500 for CHARLS and CNY 5,600 for the hospital-based study. Smoking and alcohol drinking were each categorized as never, former, or current. Sleep duration was categorized as normal sleep (6–8 h), short sleep (<6 h), and long sleep (>8 h). Number of children was grouped as 0, 1, 2, and ≥3. Employment status was grouped as no work, agricultural work, and non-agricultural work.

The covariate adjustment set was selected *a priori* based on causal considerations, prior epidemiological evidence, and the availability of harmonized variables in both data sources (22). These variables were included to reduce measured confounding rather than to represent additional exposures or study outcomes. Age and sex were included as fundamental demographic determinants of stroke susceptibility. Residence, income, and employment status were included as indicators of geographic and socioeconomic context that may be associated with residential environmental exposure patterns (23). Marital status and number of living children were included as available indicators of family structure and potential social support, which may also be related to healthcare access and stroke risk. Smoking status and alcohol drinking status were included because they are established behavioral determinants of stroke and may also vary according to socioeconomic and residential context (24). Sleep duration was included because both short and long sleep durations have been associated with stroke risk in prospective evidence (25), including a previous prospective analysis based on CHARLS (26). The coefficients of primary interest remained those for PM_2.5_, relative humidity, and the joint exposure categories.

The primary adjustment set therefore accounted for measured demographic, behavioral, socioeconomic, family-structure, and residential characteristics that could confound the associations of environmental exposure with stroke. Major vascular and other chronic conditions were evaluated in sensitivity analyses using dataset-specific comorbidity indices. The CHARLS index comprised 13 physician-diagnosed chronic conditions, including hypertension, diabetes or high blood sugar, dyslipidemia, and heart disease; atrial fibrillation was not separately ascertained in CHARLS. The hospital-based index comprised 15 pre-existing chronic conditions and separately included atrial fibrillation or other clinically documented arrhythmias. The full components and coding rules are provided in Appendix 3. These conditions were not included individually in the primary models because several were also components of the prespecified frailty index and may partly lie downstream of long-term environmental exposure. Simultaneous primary adjustment could therefore condition on components of the mediator, obscure part of the total exposure association, and introduce overadjustment. BMI was also evaluated in a sensitivity analysis because it was not uniformly available across the analytic samples; in CHARLS, relevant BMI measurements were available only in the 2011–2015 data, and valid BMI was missing for a subset of hospital-based participants.

### 2.7 Statistical analysis

Baseline characteristics were summarized according to stroke status in each data source. Categorical variables were presented as counts and proportions, and continuous variables were presented as mean and standard deviation when approximately normally distributed or as median with interquartile range when skewed. To characterize the degree of shared spatial patterning between the two environmental exposures, the correlation between continuous annual mean PM_2.5_ and continuous annual mean relative humidity was evaluated separately in each data source using Spearman rank correlation coefficients. Spearman correlation was selected because the exposure distributions were not assumed to be normally distributed and repeated exposure values could occur among participants residing in the same or nearby grid cells.

In the population-based cohort, the associations of PM_2.5_, relative humidity, and the four-category joint exposure variable with incident stroke were evaluated using Cox proportional hazards models, with results reported as hazard ratios and 95% confidence intervals. Cox regression was retained as the primary analytical approach because all participants were free of stroke at baseline and were followed prospectively, and the model allowed individual time at risk and censoring due to death, loss to follow-up, or administrative study completion to be incorporated. Person-time accrued from the baseline interview to the first follow-up interview at which stroke was reported or to the censoring date, whichever occurred first. Because the exact date of stroke onset was unavailable, the first positive follow-up interview was treated as the event-time marker; thus, this approach estimates the hazard of first stroke ascertainment and approximates the underlying onset time. In the single-center hospital study, the corresponding associations with stroke status were evaluated using multivariable logistic regression, with results reported as odds ratios and 95% confidence intervals (CIs).

All primary analyses were based on a fully adjusted model that included age, sex, marital status, residence, smoking status, alcohol drinking status, income group, sleep duration group, number of living children, and employment status. These variables were included to control for measured confounding rather than to represent additional exposures or study outcomes; the coefficients of primary interest were those for PM_2.5_, relative humidity, and the joint exposure categories. In the cohort analyses, the proportional hazards assumption was evaluated using tests based on Schoenfeld residuals. In the hospital-based logistic regression analyses, model adequacy was assessed by examining goodness of fit, multicollinearity, and influential observations. Goodness of fit was evaluated using the *Hosmer–Lemeshow* test and calibration of predicted probabilities; multicollinearity was examined using variance inflation factors; and influential observations were assessed using leverage and residual-based diagnostic measures. Discrimination of the fitted logistic models was additionally summarized using the area under the receiver operating characteristic curve. Detailed results of these model diagnostic assessments are provided in Appendix 2.

In complementary continuous-exposure analyses, annual mean PM_2.5_ and relative humidity were modeled using restricted cubic splines with three knots placed at the 10th, 50th, and 90th percentiles of the dataset-specific exposure distributions. The median exposure value in each dataset was used as the reference, and the curves were displayed over the 1st to 99th percentile range to reduce the influence of sparsely populated exposure extremes. Cox proportional hazards models were used for CHARLS, and multivariable logistic regression models were used for the hospital-based study. For each exposure-specific spline model, the other environmental exposure was included as a continuous covariate, together with the covariates included in the fully adjusted model. Overall associations were evaluated using joint Wald tests of all spline terms, whereas departures from linearity were evaluated using joint Wald tests of the nonlinear spline terms. For ease of interpretation and comparison between datasets, continuous linear estimates were also reported per 10 μg/m^3^ increase in PM_2.5_ and per 5-percentage-point increase in relative humidity. Multiplicative interaction between PM_2.5_ and relative humidity was evaluated by including the two high-versus-low exposure indicators and their product term in the fully adjusted models, consistent with the exposure definitions used in the four-category joint exposure analysis. The exponentiated coefficient for the product term was reported as the ratio of HRs in CHARLS or the ratio of ORs in the hospital-based study, together with its 95% confidence interval and corresponding *p* value for interaction. We additionally evaluated effect modification of the four-category joint exposure variable by each prespecified covariate using global multiplicative interaction tests. Stratified analyses were conducted for covariates with statistically significant interaction, and the corresponding *p* values for interaction were reported.

To evaluate frailty as a potential intermediate pathway in the associations of PM_2.5_ and relative humidity with stroke, we conducted separate regression-based counterfactual mediation analyses for each environmental exposure. The CHARLS analysis was considered the primary mediation analysis because the exposure was assigned for the year preceding baseline, frailty was assessed at baseline, and incident stroke was ascertained prospectively during follow-up. In the first analysis, PM_2.5_ was treated as the exposure and the continuous frailty index as the mediator; in the second, relative humidity was treated as the exposure and the continuous frailty index as the mediator. The mediator model regressed the frailty index on the corresponding exposure and the covariates included in the fully adjusted model using linear regression. The outcome model incorporated both the exposure and frailty and used Cox regression in CHARLS. Total associations were decomposed into natural direct and natural indirect effects, and the proportion mediated was calculated on the log-effect scale. Uncertainty was estimated using non-parametric bootstrap resampling with 5,000 iterations to obtain percentile-based 95% confidence intervals and two-sided *p* values. Causal interpretation of the natural direct and indirect effects in CHARLS requires consistency and positivity, correct specification of the mediator and outcome models, no unmeasured confounding of the exposure-outcome, exposure-mediator, or mediator-outcome relationships, no mediator-outcome confounder affected by the exposure, and appropriate temporal ordering of exposure, mediator, and outcome. Because these assumptions cannot be fully verified in observational data, the mediation estimates were interpreted as model-based evidence consistent with a potential indirect pathway rather than definitive proof of causal mediation. For the hospital-based dataset, the same regression-based decomposition was applied using logistic regression for the outcome model to provide an exploratory comparison with CHARLS. Although the frailty index was reconstructed to represent usual pre-admission or pre-stroke health status and excluded acute stroke-related deficits and in-hospital complications, the cross-sectional design cannot establish the temporal ordering of exposure, frailty, and stroke. The hospital-based direct, indirect, and mediated-proportion estimates were therefore treated as exploratory and were not assigned a causal interpretation.

The primary analyses were based on complete-case data after exclusions for missing covariates. Eight sensitivity analyses were conducted to assess robustness. First, in the cohort component, we repeated the main analyses after excluding participants who developed stroke in the first two follow-up waves to reduce the possibility of reverse causation. Second, we repeated the exposure analyses using a 2-year lag annual average exposure window instead of the primary 1-year lag window. Third, using complete-case data, the fully adjusted model was rerun with additional adjustment for body mass index (BMI, kg/m^2^). BMI was not included in the primary model because it was not uniformly available across the analytic period; in CHARLS, the relevant BMI measurements were available only in the 2011–2015 data, and valid BMI was also missing for a subset of hospital-based participants. Including BMI in the primary model would therefore have reduced the analytic sample and limited comparability with the primary analyses. This additional analysis was performed in the subset with valid BMI information to assess whether adiposity adjustment materially altered the main results. Fourth, in the CHARLS cohort, we re-estimated the associations using a prospective discrete-time logistic regression model as an alternative analytical approach because incident stroke was ascertained at follow-up waves rather than at exact event dates. As in the primary analyses, participants with stroke at baseline were excluded. The data were restructured into person-period format by survey wave, and each follow-up wave represented a discrete interval during which incident stroke could first be reported. This framework directly reflected the grouped timing of outcome ascertainment and did not require specification of an exact stroke-onset date within the interval. Follow-up time was explicitly accounted for in the model, and the fully adjusted model included the same covariates as the primary Cox regression analyses. Results from the prospective discrete-time logistic regression model were compared with those from the primary Cox models to assess whether the findings were sensitive to the choice of time-to-event framework. Fifth, we repeated the mediation analyses using categorical frailty status instead of the continuous frailty index. Sixth, to examine whether the observed associations were materially altered after accounting for baseline chronic disease burden, we additionally adjusted the fully adjusted models for a comorbidity index in both data sources. In the population-based cohort, this index was calculated from the baseline chronic disease indicators available in the dataset, excluding baseline stroke because participants with prevalent stroke had already been removed from the analytic sample. In the single-center hospital study, the comorbidity index was derived from pre-existing chronic conditions documented in the admission record, past medical history section, discharge summary, and inpatient problem list. The index was constructed as an unweighted count of chronic diseases that were explicitly recorded as having been present before or at the time of admission, such as hypertension, diabetes mellitus, dyslipidemia, coronary heart disease, heart failure, atrial fibrillation or other clinically documented arrhythmias, chronic kidney disease, chronic liver disease, chronic lung disease, malignant tumor, chronic digestive disease, arthritis or rheumatic disease, asthma, psychiatric disorder, and memory-related disease or Parkinsonism. Acute complications arising during the index hospitalization were not counted. The diagnosis of the index stroke itself was excluded by design so that the index represented background multimorbidity burden rather than the study outcome. Detailed components and coding rules of the comorbidity index are provided in Appendix 3. Seventh, to evaluate potential confounding by the broader thermal environment, we repeated the fully adjusted models with additional adjustment for annual mean ambient temperature. Temperature was obtained from ERA5 and assigned using the same residential location and one-year lagged annual average exposure window as relative humidity. Annual mean temperature was modeled as a continuous covariate. Finally, to evaluate potential broad regional confounding and correlation among participants residing within the same geographic area, we repeated the primary CHARLS analyses using province-level fixed effects and robust sandwich variance estimates clustered at the residential-community level. Province-level fixed effects were used to control for broad differences in climatic conditions, healthcare context, socioeconomic development, and other spatially patterned characteristics, whereas community-clustered variance estimates accounted for statistical correlation among participants residing in the same community. This analysis was restricted to CHARLS because residential-community identifiers and sufficient province-level variation were available in that dataset. An equivalent analysis was not performed in the hospital-based study because consistent community-level cluster identifiers were unavailable and most participants resided in Fujian Province, providing insufficient between-province variation for stable province-level estimation.

All statistical analyses and figures were performed using Stata/SE 17.0 (StataCorp LLC, College Station, TX, United States) and R (version 4.0.5; R Core Team, 2020). All tests were two-sided, and *p* < 0.05 was considered statistically significant.

### 2.8 Ethics

The population-based cohort component was approved by the Biomedical Ethics Review Committee at Peking University (IRB00001052-11015), and all participants provided written informed consent. The single-center hospital study was approved by the Ethics Committee of Jinjiang Municipal Hospital (Shanghai Sixth People’s Hospital Fujian) (approval no. jjsyyll-2026-044). The study was conducted in accordance with the Declaration of Helsinki, and all data were de-identified before analysis.

## 3 Results

### 3.1 Characteristics of included participants

Table 1 summarizes the baseline characteristics of participants in CHARLS and the single-center hospital-based study by stroke status. The final analytic sample comprised 17,197 participants in CHARLS, among whom 1,485 developed incident stroke during follow-up, and 782 participants in the hospital-based study, including 148 stroke patients and 634 non-stroke controls. Among the 148 hospital-based stroke cases, 117 (79.1%) were classified as ischemic stroke, 26 (17.6%) as intracerebral hemorrhage, and 5 (3.4%) as subarachnoid hemorrhage. Stroke subtype was not available in CHARLS.

**Table 1:** Baseline characteristics of participants by incident stroke status in CHARLS and by stroke status in the single-center hospital-based study.

Characteristics	CHARLS			Hospital-based study		
	Total (*N* = 17,197)	No incident stroke (*N* = 15,712)	Incident stroke (*N* = 1,485)	Total (*N* = 782)	Non-stroke (*N* = 634)	Stroke (*N* = 148)
Age (years)	57.32 (9.80)	57.28 (9.90)	57.78 (8.73)	69.40 (8.10)	68.50 (7.90)	73.10 (8.30)
Sex (*n*, %)						
Female	8,939 (52.0)	8,186 (52.1)	753 (50.7)	410 (52.4)	351 (55.4)	59 (39.9)
Male	8,258 (48.0)	7,526 (47.9)	732 (49.3)	372 (47.6)	283 (44.6)	89 (60.1)
Marital status (*n*, %)						
Other/unknown	1,913 (11.1)	1,759 (11.2)	154 (10.4)	298 (38.1)	223 (35.2)	75 (50.7)
Married	15,284 (88.9)	13,953 (88.8)	1,331 (89.6)	484 (61.9)	411 (64.8)	73 (49.3)
Residence (*n*, %)						
Rural	10,640 (61.9)	9,762 (62.1)	878 (59.1)	385 (49.2)	330 (52.1)	55 (37.2)
Urban	6,557 (38.1)	5,950 (37.9)	607 (40.9)	397 (50.8)	304 (47.9)	93 (62.8)
Smoking status (*n*, %)						
Never smoking	10,545 (61.3)	9,663 (61.5)	882 (59.4)	413 (52.8)	358 (56.5)	55 (37.2)
Former smoking	1,524 (8.9)	1,375 (8.8)	149 (10.0)	157 (20.1)	118 (18.6)	39 (26.4)
Current smoking	5,128 (29.8)	4,674 (29.7)	454 (30.6)	212 (27.1)	158 (24.9)	54 (36.5)
Alcohol drinking status (*n*, %)						
Never drinking	9,263 (53.9)	8,457 (53.8)	806 (54.3)	393 (50.3)	330 (52.1)	63 (42.6)
Former drinking	1,521 (8.8)	1,376 (8.8)	145 (9.8)	98 (12.5)	72 (11.4)	26 (17.6)
Current drinking	6,413 (37.3)	5,879 (37.4)	534 (36.0)	291 (37.2)	232 (36.6)	59 (39.9)
Income group (*n*, %)						
Below median	12,863 (74.8)	11,758 (74.8)	1,105 (74.4)	461 (58.9)	352 (55.5)	109 (73.6)
At or above median	4,334 (25.2)	3,954 (25.2)	380 (25.6)	321 (41.1)	282 (44.5)	39 (26.4)
Sleep duration group (*n*, %)						
Normal sleep (6–8 h)	7,777 (45.2)	7,140 (45.4)	637 (42.9)	478 (61.1)	414 (65.3)	64 (43.2)
Short sleep (<6 h)	8,799 (51.2)	8,013 (51.0)	786 (52.9)	233 (29.8)	172 (27.1)	61 (41.2)
Long sleep (>8 h)	621 (3.6)	559 (3.6)	62 (4.2)	71 (9.1)	48 (7.6)	23 (15.5)
Number of children (*n*, %)						
0	375 (2.2)	355 (2.3)	20 (1.3)	83 (10.6)	74 (11.7)	9 (6.1)
1	3,285 (19.1)	3,020 (19.2)	265 (17.8)	154 (19.7)	132 (20.8)	22 (14.9)
2	6,361 (37.0)	5,815 (37.0)	546 (36.8)	284 (36.3)	238 (37.4)	46 (31.1)
≥3	7,176 (41.7)	6,522 (41.5)	654 (44.0)	261 (33.4)	190 (30.1)	71 (48.0)
Employment status (*n*, %)						
No work	5,604 (32.6)	5,069 (32.3)	535 (36.0)	374 (47.8)	273 (43.1)	101 (68.2)
Agricultural work	6,669 (38.8)	6,104 (38.8)	565 (38.0)	204 (26.1)	176 (27.8)	28 (18.9)
Non-agricultural work	4,924 (28.6)	4,539 (28.9)	385 (25.9)	204 (26.1)	185 (29.2)	19 (12.8)
Frailty group (*n*, %)						
Non-frail	15,494 (90.1)	14,214 (90.5)	1,280 (86.2)	553 (70.7)	492 (77.6)	61 (41.2)
Frail	1,703 (9.9)	1,498 (9.5)	205 (13.8)	229 (29.3)	142 (22.4)	87 (58.8)
PM_2.5_(μg/m^³^)	53.21 (16.74)	52.91 (16.71)	56.33 (16.67)	29.01 (8.91)	27.61 (8.40)	34.81 (9.20)
Relative humidity (%)	66.59 (8.50)	66.41 (8.47)	68.47 (8.63)	73.91 (8.51)	72.71 (8.10)	79.01 (8.31)
PM_2.5_ group (*n*, %)						
Low	7,188 (41.8)	6,674 (42.5)	514 (34.6)	392 (50.1)	352 (55.5)	40 (27.0)
High	10,009 (58.2)	9,038 (57.5)	971 (65.4)	390 (49.9)	282 (44.5)	108 (73.0)
Relative humidity group (*n*, %)						
Low	9,499 (55.2)	8,767 (55.8)	732 (49.3)	392 (50.1)	353 (55.7)	39 (26.4)
High	7,698 (44.8)	6,945 (44.2)	753 (50.7)	390 (49.9)	281 (44.3)	109 (73.6)
Joint exposure (*n*, %)						
Low PM_2.5_ and low humidity	3,177 (18.5)	3,012 (19.2)	165 (11.1)	281 (35.9)	268 (42.3)	13 (8.8)
High PM_2.5_ and low humidity	6,322 (36.8)	5,755 (36.6)	567 (38.2)	111 (14.2)	85 (13.4)	26 (17.6)
Low PM_2.5_ and high humidity	4,011 (23.3)	3,662 (23.3)	349 (23.5)	111 (14.2)	84 (13.2)	27 (18.2)
High PM_2.5_ and high humidity	3,687 (21.4)	3,283 (20.9)	404 (27.2)	279 (35.7)	197 (31.1)	82 (55.4)
Frailty index	0.13 (0.09)	0.13 (0.09)	0.15 (0.09)	0.19 (0.11)	0.18 (0.10)	0.21 (0.12)

Values are presented as mean (SD) for continuous variables and *n* (column proportion) for categorical variables. In CHARLS, stroke status denotes incident stroke occurring during follow-up among participants free of stroke at baseline; in the single-center hospital-based study, stroke status denotes stroke versus non-stroke at the index hospitalization. Annual mean PM_2.5_ and relative humidity exposures were assigned according to residential location for the year immediately preceding the baseline interview in CHARLS or the index date in the hospital-based study. PM_2.5_ group and relative humidity group were defined using the database-specific median of the corresponding annual mean exposure. Joint exposure was defined by cross-classification of PM_2.5_ and relative humidity into four categories: low PM_2.5_-low humidity, high PM_2.5_-low humidity, low PM_2.5_-high humidity, and high PM_2.5_-high humidity. Frailty group was derived from the deficit-accumulation frailty index, with frailty defined as FI ≥0.25. Income group was categorized as below median and at or above median using the study-specific median; in CHARLS, the cut-off was based on the non-zero median. The corresponding cut-off values were CNY 4,500 in CHARLS and CNY 5,600 in the hospital-based study. Sleep duration was categorized as normal sleep (6–8 h), short sleep (<6 h), and long sleep (>8 h). Number of children was grouped as 0, 1, 2, and ≥3. Percentages may not sum to 100 because of rounding.

In CHARLS, participants who developed incident stroke tended to be slightly older than those who remained stroke-free. Compared with participants without incident stroke, those who developed stroke were modestly more likely to be male, married, and living in urban areas. In terms of health-related behaviors, the stroke group showed a lower proportion of never smokers and a slightly higher proportion of former and current smokers. Alcohol-use patterns were less distinct, although participants who developed stroke showed somewhat higher proportions of never and former drinkers and a slightly lower proportion of current drinkers. Income distribution was broadly similar between the two groups.

Sleep duration patterns also differed modestly by stroke status. Participants who developed stroke were more likely to report short sleep and long sleep, and less likely to report normal sleep. They were also more likely to have three or more children and to be without work, whereas non-agricultural work was less common. Environmental exposure profiles were also less favorable in the stroke group, with higher annual mean PM_2.5_ and higher annual mean relative humidity. Consistently, they were more likely to be classified in the high PM_2.5_ group and the high humidity group. In the joint exposure classification, participants who developed stroke were less likely to fall into the low PM_2.5_–low humidity category and more likely to be in the high PM_2.5_–low humidity and especially the high PM_2.5_–high humidity category, whereas the low PM_2.5_–high humidity category was broadly similar between groups.

In the single-center hospital-based study, stroke patients were notably older than non-stroke controls and were more likely to be male. They were also more likely to have other or unknown marital status and to live in urban areas. Health-behavior profiles differed more clearly than in CHARLS: compared with non-stroke controls, stroke patients were less likely to be never smokers and more likely to be former or current smokers. A similar pattern was observed for alcohol use, with stroke patients being less likely to be never drinkers and more likely to be former or current drinkers. In contrast to CHARLS, stroke patients in the hospital dataset were more likely to belong to the lower income group.

Sleep duration showed clearer separation in the hospital sample. Stroke patients were less likely to report normal sleep and more likely to report short sleep or long sleep. They were also more likely to have three or more children and to be without work, while both agricultural and non-agricultural work were less common. Environmental exposure patterns were also more distinct. Stroke patients had higher annual mean PM_2.5_ and higher relative humidity and were much more likely to be classified in the high PM_2.5_ and high humidity groups. In the joint exposure classification, they were far less likely to be in the low PM_2.5_–low humidity category and much more likely to be in the high PM_2.5_–high humidity category, with smaller but still higher proportions also observed for the high PM_2.5_–low humidity and low PM_2.5_–high humidity categories.

The spatial relationship between the two continuous environmental exposures differed between the data sources. In CHARLS, annual mean PM_2.5_ and annual mean relative humidity showed a weak inverse correlation (Spearman ρ = −0.214, *p* < 0.001). In the single-center hospital-based study, the two exposures showed a moderate positive correlation (Spearman ρ = 0.438, *p* < 0.001). Although some shared spatial patterning was present, particularly in the geographically concentrated hospital sample, the magnitude of correlation did not indicate that PM_2.5_ and relative humidity represented redundant exposure measures.

### 3.2 Associations of PM_2.5_ and ambient humidity exposures with stroke

**Figure 2:**
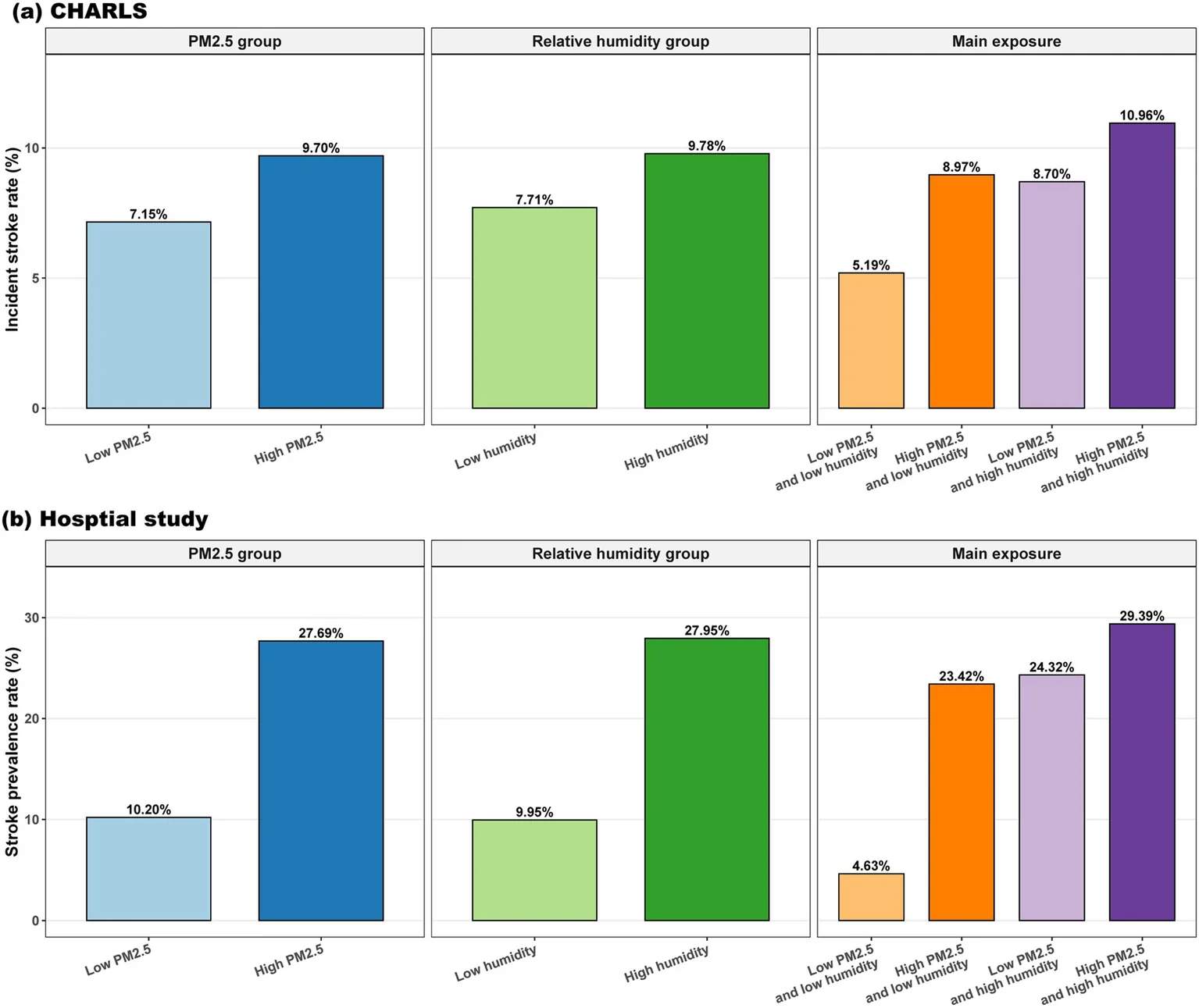
Crude stroke occurrence across PM_2.5_, relative humidity, and their joint exposure categories in CHARLS and the single-center hospital-based study. Panel **(a)** shows the incident stroke rate (%) in CHARLS, and panel **(b)** shows the stroke prevalence (%) in the single-center hospital-based study. Within each panel, the left subplot presents stroke occurrence according to the PM_2.5_ group (low vs. high), the middle subplot presents stroke occurrence according to the relative humidity group (low vs. high), and the right subplot presents stroke occurrence across the four-category joint exposure classification: Low PM_2.5_ and low humidity, high PM_2.5_ and low humidity, low PM_2.5_ and high humidity, and high PM_2.5_ and high humidity. Low exposure was defined as a value below the dataset-specific median, and high exposure was defined as a value equal to or above the median. The cut-off values were 52.1 μg/m^3^ for PM_2.5_ and 67.0% for relative humidity in CHARLS, and 28.8 μg/m^3^ and 74.2%, respectively, in the single-center hospital-based study. Bars represent the proportion of stroke within each exposure category, and the values shown above the bars indicate the corresponding percentages.

Figure 2 presents the stroke occurrence patterns across PM_2.5_, relative humidity, and their joint exposure categories in CHARLS and the single-center hospital-based study. In CHARLS, the incident stroke rate was higher in the high PM_2.5_ group than in the low PM_2.5_ group (9.70% vs. 7.15%) and was also higher in the high humidity group than in the low humidity group (9.78% vs. 7.71%). In the joint exposure categories, the lowest incident stroke rate was observed in the low PM_2.5_ and low humidity group (5.19%), while the highest was observed in the high PM_2.5_ and high humidity group (10.96%); intermediate rates were seen for high PM_2.5_ and low humidity (8.97%) and low PM_2.5_ and high humidity (8.70%). A similar but more pronounced pattern was observed in the hospital-based study. Stroke prevalence was substantially higher in the high PM_2.5_ group than in the low PM_2.5_ group (27.69% vs. 10.20%) and in the high humidity group than in the low humidity group (27.95% vs. 9.95%). Across the four joint exposure categories, prevalence was lowest in the low PM_2.5_ and low humidity group (4.63%) and highest in the high PM_2.5_ and high humidity group (29.39%), with elevated values also observed for high PM_2.5_ and low humidity (23.42%) and low PM_2.5_ and high humidity (24.32%).

**Figure 3:**
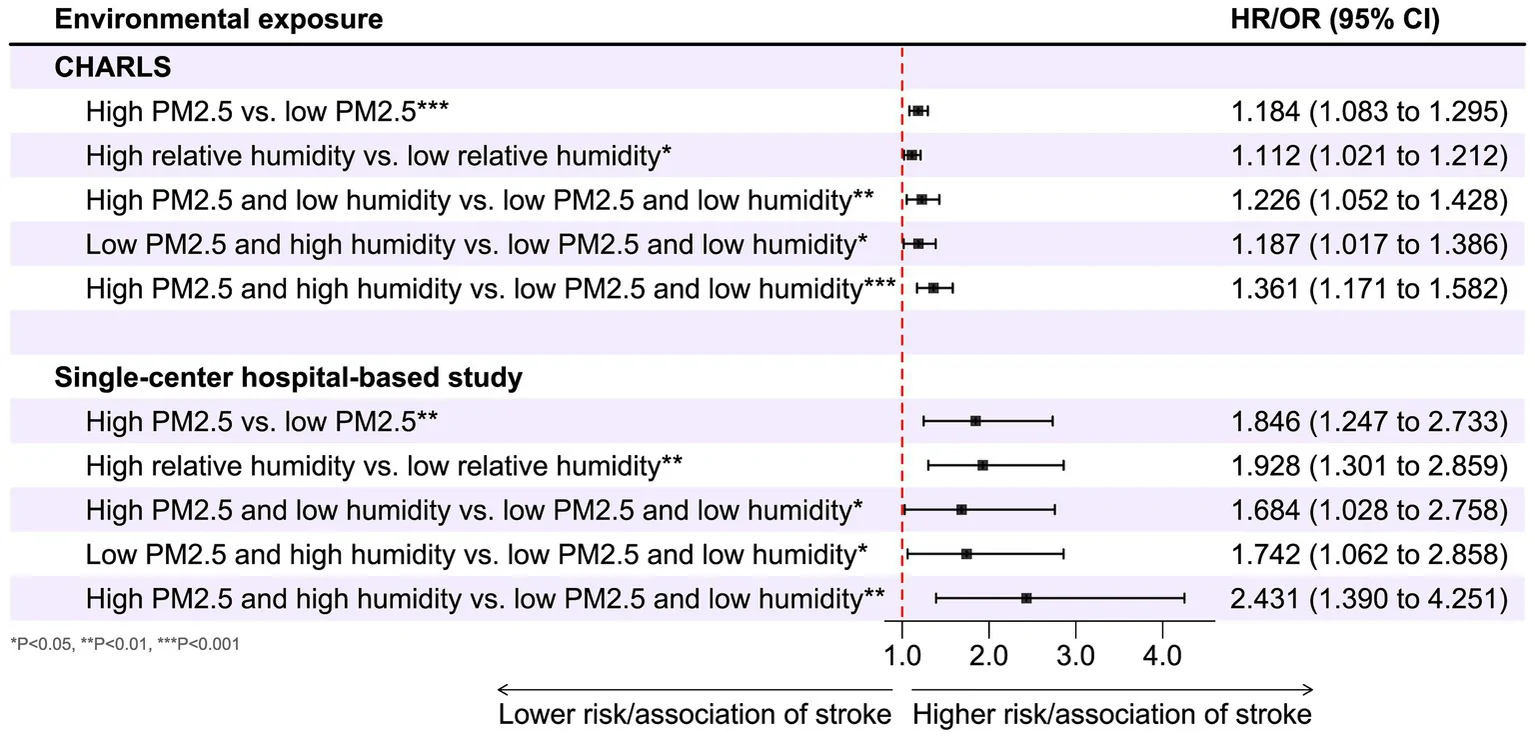
Associations of PM_2.5_ exposure, ambient relative humidity, and their joint exposure categories with stroke in CHARLS and the single-center hospital-based study. Results are shown as hazard ratios (HRs) with 95% confidence intervals (CIs) for CHARLS and as odds ratios (ORs) with 95% CIs for the single-center hospital-based study. For the single-exposure analyses, the comparisons are high PM_2.5_ versus low PM_2.5_ and high relative humidity versus low relative humidity. For the joint exposure analyses, the reference group is low PM_2.5_ and low humidity, and the three comparison groups are high PM_2.5_ and low humidity, low PM_2.5_ and high humidity, and high PM_2.5_ and high humidity. The vertical dashed line indicates the null value (HR/OR = 1.0). All estimates were adjusted for age, sex, marital status, residence, smoking status, alcohol drinking status, income group, sleep duration group, number of children, and employment status. ^*^p < 0.05, ^**^p < 0.01, ^***^p < 0.001.

Figure 3 summarizes the associations of PM_2.5_ exposure, ambient relative humidity, and their joint exposure categories with stroke in CHARLS and the single-center hospital-based study. In the fully adjusted models, both higher PM_2.5_ and higher relative humidity were associated with less favorable stroke outcomes, and the joint exposure analyses suggested that the greatest elevations were observed when high PM_2.5_ and high humidity co-occurred.

In CHARLS, compared with the low PM_2.5_ group, participants in the high PM_2.5_ group had a higher risk of incident stroke (HR = 1.184, 95% CI 1.083–1.295). Similarly, high relative humidity was associated with a higher incident stroke risk compared with low relative humidity (HR = 1.112, 95% CI 1.021–1.212). In the joint exposure model, using low PM_2.5_ and low humidity as the reference category, incident stroke risk was higher for high PM_2.5_ and low humidity (HR = 1.226, 95% CI 1.052–1.428) and for low PM_2.5_ and high humidity (HR = 1.187, 95% CI 1.017–1.386). The largest association was observed for the combined exposure of high PM_2.5_ and high humidity, with a hazard ratio of 1.361 (95% CI 1.171–1.582). Overall, these findings indicate that in the population-based cohort, both environmental factors were independently associated with higher stroke risk, with additional elevation when both exposures were present simultaneously.

In the single-center hospital-based study, the direction of associations was consistent but effect sizes were larger. Compared with low PM_2.5_, high PM_2.5_ was associated with higher odds of stroke (OR = 1.846, 95% CI 1.247–2.733), and high relative humidity was likewise associated with higher stroke odds (OR = 1.928, 95% CI 1.301–2.859). In the joint exposure analysis, relative to low PM_2.5_ and low humidity, the odds of stroke were increased for high PM_2.5_ and low humidity (OR = 1.684, 95% CI 1.028–2.758) and for low PM_2.5_ and high humidity (OR = 1.742, 95% CI 1.062–2.858). As in CHARLS, the highest effect estimate was observed for high PM_2.5_ and high humidity, which was associated with markedly higher odds of stroke (OR = 2.431, 95% CI 1.390–4.251).

**Figure 4:**
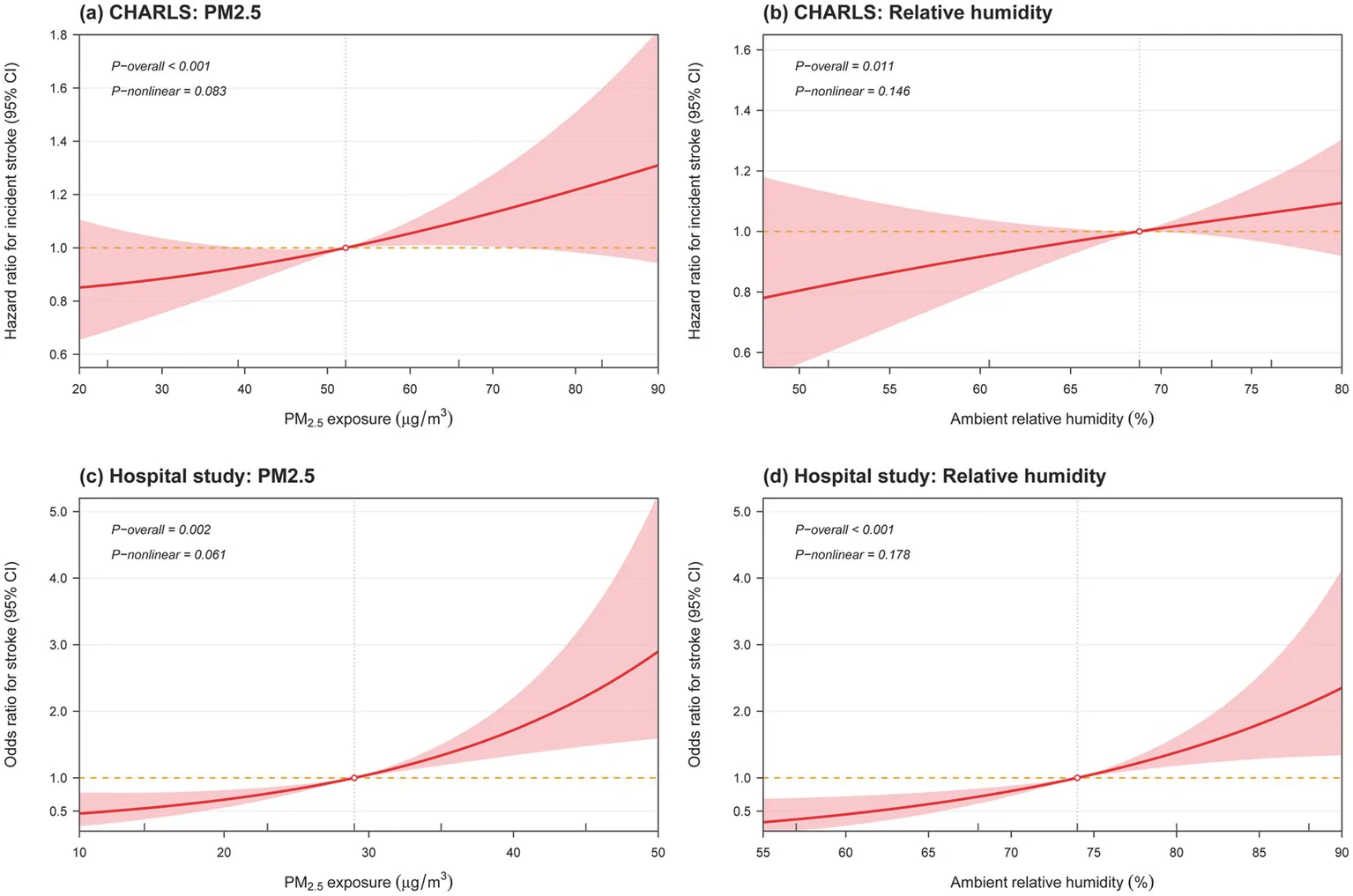
Panel **(a)** shows PM2.5 and incident stroke in CHARLS; panel **(b)** shows relative humidity and incident stroke in CHARLS; panel **(c)** shows PM_2.5_ and stroke status in the single-center hospital-based study; and panel **(d)** shows relative humidity and stroke status in the single-center hospital-based study. Three knots were placed at the 10th, 50th, and 90th percentiles of each database-specific exposure distribution. The median exposure value was used as the reference, indicated by the vertical dotted line, and the horizontal dashed line represents HR/OR = 1.0. Curves were displayed from the 1st to the 99th percentile. Models were adjusted for age, sex, marital status, residence, smoking status, alcohol drinking status, income group, sleep duration group, number of living children, employment status, and the other continuous environmental exposure. P-overall evaluates the overall exposure association, and P-nonlinear evaluates departure from linearity.

Continuous-exposure analyses were consistent with the primary categorical findings. In CHARLS, each 10 μg/m^3^ increase in annual mean PM_2.5_ was associated with a 7.2% higher hazard of incident stroke (HR 1.072, 95% CI 1.042–1.103), and each 5-percentage-point increase in relative humidity was associated with a 3.6% higher hazard (HR 1.036, 95% CI 1.008–1.065). In the hospital-based study, the corresponding estimates were OR 1.431 (95% CI 1.146–1.787) for PM_2.5_ and OR 1.218 (95% CI 1.086–1.366) for relative humidity. Restricted cubic spline analyses showed generally increasing exposure-response patterns, without statistically clear evidence of nonlinearity in CHARLS or the hospital-based study (all *P* for nonlinearity >0.05; Figure 4).

Formal multiplicative interaction testing did not identify statistically significant interaction between the dichotomized PM_2.5_ and relative humidity exposures. The interaction ratios were 0.935 (95% CI 0.770–1.136; *P* for interaction = 0.500) in CHARLS and 0.829 (95% CI 0.413–1.662; *P* for interaction = 0.597) in the hospital-based study. Thus, although the joint high PM_2.5_-high humidity category consistently showed the largest effect estimate, there was no evidence that the combined association exceeded that expected on the multiplicative scale.

### 3.3 Model-based mediation analyses involving frailty

**Figure 5:**
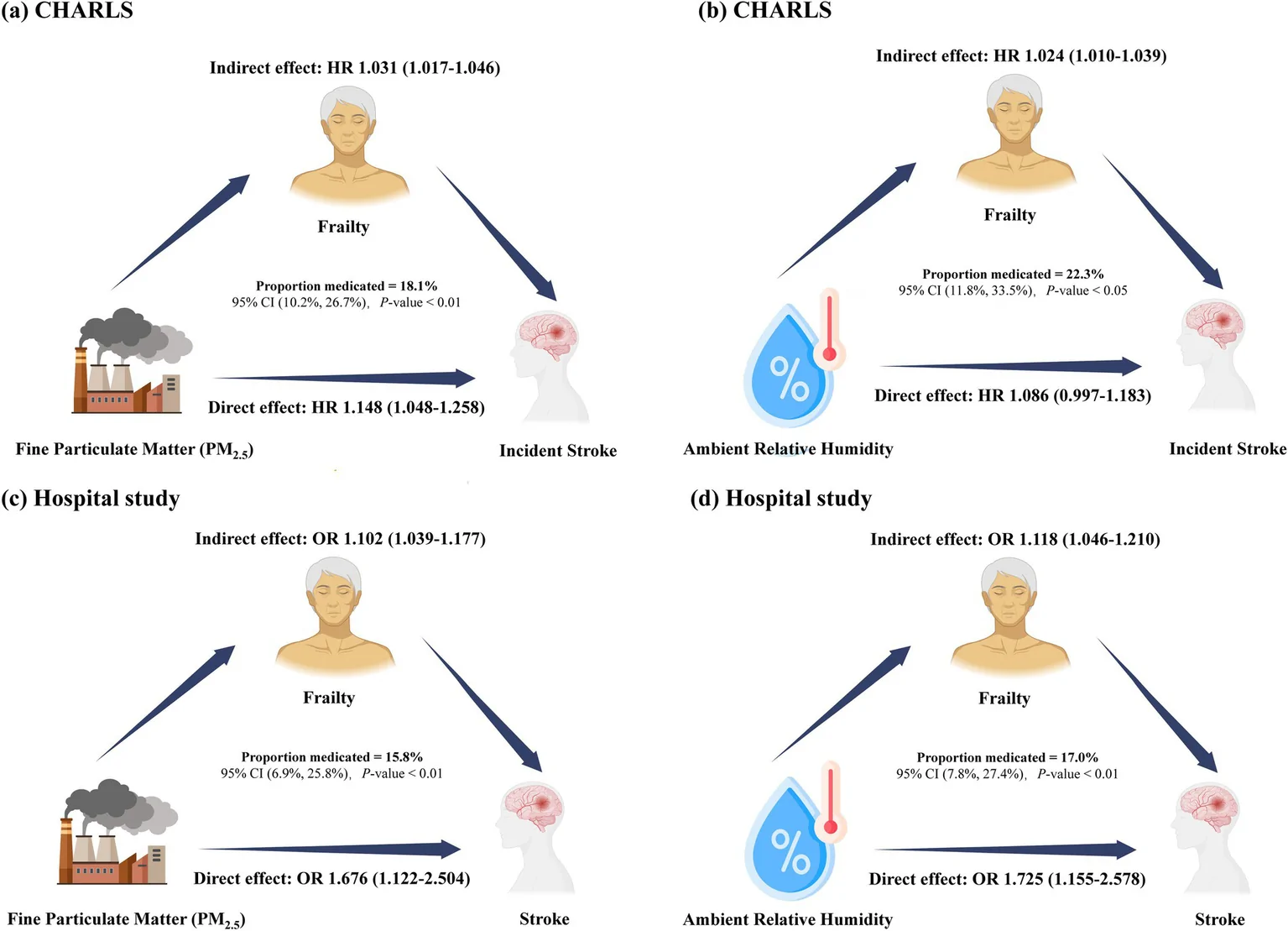
Model-based mediation analyses involving frailty for PM_2.5_, ambient relative humidity, and stroke in CHARLS and the single-center hospital-based study. Exposures were defined as high versus low PM_2.5_ or relative humidity using database-specific medians of the annual mean values in the year preceding baseline or the hospital index date. Frailty was modeled as a continuous deficit-accumulation index. Panels **(a,b)** present the primary CHARLS analyses for incident stroke, whereas panels **(c,d)** present exploratory analyses of stroke status in the cross-sectional hospital-based sample. Each panel shows the direct-effect estimate, the indirect-effect estimate involving frailty, and the estimated proportion mediated. Models were adjusted for age, sex, marital status, residence, smoking, alcohol use, income, sleep duration, number of living children, and employment status; 95% confidence intervals were obtained from 5,000 bootstrap resamples. The CHARLS estimates should be interpreted subject to the identification assumptions described in the methods. The hospital-based estimates are exploratory and do not establish temporal or causal mediation.

Figure 5 summarizes the model-based decomposition of the associations of PM_2.5_ and ambient relative humidity with stroke into total, direct, and frailty-related indirect effects in CHARLS and the single-center hospital-based study. In the primary CHARLS mediation analyses, the model-based natural indirect effect of high PM_2.5_ on incident stroke involving frailty was statistically significant (HR 1.031, 95% CI 1.017–1.046), corresponding to an estimated mediated proportion of 18.1% (95% CI 10.2–26.7%). Under the identification assumptions described in the Methods, this pattern was consistent with a potential partial indirect pathway involving frailty. The corresponding natural direct effect was HR 1.148 (95% CI 1.048–1.258), and the total effect was HR 1.184 (95% CI 1.083–1.295), consistent with the fully adjusted primary model. For high relative humidity, the model-based natural indirect effect involving frailty was also statistically significant (HR 1.024, 95% CI 1.010–1.039), corresponding to an estimated mediated proportion of 22.3% (95% CI 11.8–33.5%). The corresponding natural direct effect was HR 1.086 (95% CI 0.997–1.183), and the total effect was HR 1.112 (95% CI 1.021–1.212). These estimates should be interpreted in the context of the causal identification assumptions stated in the Methods, which cannot be fully verified in observational data.

In the exploratory single-center hospital-based analysis, application of the same model-based decomposition yielded indirect-effect estimates in the same direction as those observed in CHARLS. For high PM_2.5_, the model-based indirect effect involving frailty was statistically significant (OR 1.102, 95% CI 1.039–1.177), corresponding to an estimated mediated proportion of 15.8% (95% CI 6.9–25.8%). The corresponding direct-effect estimate was OR 1.676 (95% CI 1.122–2.504), and the total-effect estimate was OR 1.846 (95% CI 1.247–2.733), consistent with the fully adjusted primary model. For high relative humidity, the model-based indirect-effect estimate involving frailty was OR 1.118 (95% CI 1.046–1.210), with an estimated mediated proportion of 17.0% (95% CI 7.8–27.4%). The corresponding direct-effect estimate was OR 1.725 (95% CI 1.155–2.578), and the total-effect estimate was OR 1.928 (95% CI 1.301–2.859). Because environmental exposure, reconstructed pre-admission frailty, and stroke status were evaluated within a cross-sectional clinical design, these hospital-based estimates are exploratory and should not be interpreted as establishing temporal or causal mediation.

### 3.4 Subgroup analyses by sex and employment status

Among all prespecified covariates evaluated using global multiplicative interaction tests, sex and employment status were the only variables for which interaction with the four-category joint PM_2.5_–humidity exposure was statistically significant in both datasets. For sex, the *p* values for interaction were 0.021 in CHARLS and <0.001 in the single-center hospital-based study; for employment status, the corresponding *p* values were <0.001 and 0.003. Across both datasets, the associations between the joint exposure categories and stroke were generally stronger among women than among men and among participants without work than among those engaged in agricultural or non-agricultural work. In CHARLS, the high PM_2.5_ and high humidity category was associated with an HR of 1.548 (95% CI 1.234–1.942) among women and 1.247 (95% CI 1.001–1.554) among men; among participants without work, the corresponding HR was 1.664 (95% CI 1.294–2.140). A similar pattern was observed in the hospital-based study, in which the high PM_2.5_ and high humidity category was associated with an OR of 3.012 (95% CI 1.368–6.631) among women, 2.203 (95% CI 1.061–4.577) among men, and 3.284 (95% CI 1.575–6.845) among participants without work. In nearly all subgroup comparisons, the high PM_2.5_ and high humidity category yielded the largest effect estimate relative to the low PM_2.5_ and low humidity reference group. Detailed stratum-specific estimates and interpretation of the subgroup analyses are provided in Appendix 4.

### 3.5 Sensitivity analyses

Eight sensitivity analyses supported the robustness of the primary findings. First, in the CHARLS cohort, exclusion of participants who developed stroke in the first two follow-up waves yielded effect estimates that remained directionally consistent with the primary results (Appendix 5). Second, redefining the exposure window using a 2-year lag annual average instead of the primary 1-year lag produced similar associations in both data sources (Appendix 6). Third, additional adjustment for BMI in the subset with valid BMI information did not materially alter the results (Appendix 7). Fourth, in CHARLS, the prospective discrete-time logistic regression model yielded estimates that were closely comparable to those from the primary Cox models. The discrete-time ORs were 1.189 (95% CI 1.087–1.302) for high PM_2.5_, 1.118 (95% CI 1.026–1.220) for high relative humidity, and 1.371 (95% CI 1.177–1.596) for joint high PM_2.5_ and high humidity; the corresponding Cox HRs were 1.184, 1.112, and 1.361, respectively (Appendix 8). Fifth, repeating the mediation analyses using categorical frailty status instead of the continuous frailty index produced directionally consistent findings (Appendix 9). Sixth, further adjustment for the comorbidity index in both data sources resulted in only modest attenuation of the estimates, with the overall pattern of association unchanged (Appendix 10). Seventh, additional adjustment for annual mean ambient temperature produced modest attenuation, particularly for the humidity-related estimates, but did not materially change the overall pattern. In CHARLS, the temperature-adjusted HRs were 1.176 (95% CI 1.073–1.289) for high PM_2.5_, 1.098 (95% CI 1.005–1.199) for high relative humidity, and 1.327 (95% CI 1.141–1.543) for the high PM_2.5_ and high humidity category. In the hospital-based study, the corresponding ORs were 1.792 (95% CI 1.199–2.678), 1.741 (95% CI 1.140–2.659), and 2.216 (95% CI 1.238–3.966), respectively. The high PM_2.5_ and high humidity category remained statistically significant and retained the largest effect estimate in both datasets (Appendix 11). Eighth, CHARLS participants were distributed across 450 residential communities in 28 provincial-level regions, with marked regional variation in annual mean PM_2.5_, relative humidity, and ambient temperature. In spatially adjusted Cox models incorporating province-level fixed effects and community-clustered robust variance estimates, high PM_2.5_, high relative humidity, and the joint high PM_2.5_-high humidity category remained positively associated with incident stroke. The joint high-exposure category retained the largest effect estimate (HR 1.298, 95% CI 1.084–1.554), although the two intermediate joint exposure contrasts were attenuated and no longer statistically significant (Appendix 12). Overall, these analyses indicated that alternative sample restrictions, exposure windows, time-to-event specifications, mediator definitions, adjustment for BMI and baseline multimorbidity, control for annual mean ambient temperature, and consideration of broad province-level differences and within-community correlation did not materially alter the principal conclusions. Detailed results and interpretation of all sensitivity analyses are provided in Appendix 13.

## 4 Discussion

In this study, higher PM_2.5_ exposure and higher ambient relative humidity were each associated with less favorable stroke outcomes in both the nationally based CHARLS cohort and the single-center hospital-based sample. The joint exposure analyses showed the same ordering in both datasets, with the lowest risk in the low PM_2.5_ and low humidity group and the largest effect estimates in the high PM_2.5_ and high humidity group. Model-based mediation analyses suggested that frailty may account for a nontrivial proportion of these associations, with estimated mediated proportions of 18.1% for PM_2.5_ and 22.3% for humidity in CHARLS; the corresponding exploratory estimates in the hospital-based study were 15.8 and 17.0%, respectively. The subgroup analyses also pointed to stronger associations among women and among participants without work. To our knowledge, this study provides the first evidence integrating a population-based prospective cohort and a clinical hospital-based sample to examine the joint associations of PM_2.5_ and annual average ambient humidity with stroke while also evaluating frailty as a potential mediator.

The PM_2.5_ findings are broadly consistent with recent evidence from other settings, although the magnitude of association differs across populations and exposure metrics. In a national cohort study of 17,443,900 older Medicare beneficiaries in the United States, Ma et al. identified about 2.2 million incident stroke cases and found that an interquartile range increase in annual PM_2.5_, defined as 3.7 μg/m^3^, was associated with an HR of 1.022, 95% CI 1.017 to 1.028, for incident stroke (27). In another nationwide analysis of American older adults, Jin et al. followed Medicare enrollees aged 65 years or older and reported that each 1 μg/m^3^ increase in annual PM_2.5_ was associated with an HR of 1.0279, 95% CI 1.0274 to 1.0284, for stroke incidence (28). Evidence from Europe has also suggested that susceptibility is not evenly distributed. In a Danish cohort of 1,971,246 adults aged 50 to 85 years, including 83,211 incident stroke cases, Poulsen et al. observed stronger PM_2.5_ associated stroke risks among participants with lower educational attainment and lower income, with an HR of 1.28, 95% CI 1.22 to 1.34, in those with mandatory education (29). In China, Fang et al. analyzed 13,573 middle aged and older adults from CHARLS and reported that each 10 μg/m^3^ increase in PM_2.5_ was associated with an OR of 1.05 for incident stroke, with stronger associations among women and among participants who were unemployed or engaged in agricultural work (30). Compared with those studies, the present analysis adds evidence on annual average ambient humidity, shows that PM_2.5_ and humidity jointly identify a group with particularly elevated stroke risk, and demonstrates that the direction of association is reproducible across both a community-based cohort and a clinical sample. In addition, the atmospheric relationship between relative humidity and PM_2.5_ is complex and should not be assumed to be uniformly inverse. Precipitation can remove particulate matter through wet scavenging, but high relative humidity alone does not necessarily indicate precipitation or effective particle removal. Under non-precipitating or stagnant conditions, higher relative humidity can promote aerosol water uptake, hygroscopic growth, gas–particle partitioning, multiphase reactions, and secondary aerosol formation (31–33). The net relationship therefore varies according to precipitation, atmospheric ventilation, temperature, aerosol composition, precursor emissions, season, and geographic setting. Consistent with this heterogeneity, PM_2.5_ and relative humidity showed a weak inverse correlation in CHARLS but a moderate positive correlation in the geographically concentrated hospital-based sample. The high PM_2.5_–high humidity category should therefore be interpreted as an empirically observed joint exposure group rather than as evidence that humidity caused higher PM_2.5_ concentrations. Moreover, because the formal multiplicative interaction tests were not statistically significant, the larger estimate in the high–high category does not establish synergistic interaction between the two exposures.

The model-based mediation analyses suggest that frailty may be one pathway through which environmental exposure becomes biologically and clinically relevant for stroke. Frailty is not simply a count of chronic illnesses, but a broader indicator of reduced reserve across physical, functional, and psychosocial domains, and that makes it a plausible intermediate phenotype linking environmental stressors to cerebrovascular vulnerability. A recent meta-analysis by Burton et al. pooled 14 studies with 27,210 patients with acute stroke and estimated that pre-stroke frailty was present in 24.6, 95% CI 16.2 to 33.1, while also showing that frailty was associated with markedly higher longer-term mortality after stroke, with an OR of 3.75, 95% CI 2.41 to 5.70 (34). Evidence also suggests that frailty itself is environmentally patterned. In the Korean Frailty and Aging Cohort Study, Shin and Choi analyzed 2,912 community-dwelling older adults and found that each 1 μg/m^3^ increase in PM_2.5_ was associated with an OR of 1.055, 95% CI 1.002 to 1.112, for frailty and an OR of 1.053, 95% CI 1.017 to 1.090, for pre-frailty (35). More recently, Jafari et al. synthesized 18 studies and reported a pooled OR of 1.19, 95% CI 1.10 to 1.27, for the association between PM_2.5_ and frailty across nine studies included in the quantitative meta-analysis (36). Collectively, these studies provide comparatively direct epidemiological support for a potential PM_2.5_-frailty pathway. By contrast, direct epidemiological evidence specifically linking long-term ambient relative humidity to frailty remains sparse. The available support is primarily indirect. A national longitudinal study reported an association between heatwave exposure and a higher incidence of frailty, while experimental evidence suggests that older adults may accumulate greater body heat than younger adults during both dry and humid heat exposure. Together with physiological evidence that higher humidity can restrict evaporative cooling and increase thermoregulatory, cardiovascular, and fluid-regulatory demands, these observations support a broader thermal-stress pathway through which recurrent environmental strain could contribute to exhaustion, functional decline, and the accumulation of health deficits (10, 15, 37, 38). However, heatwaves and acute humid-heat exposure are not equivalent to annual average relative humidity, and these studies do not establish a direct long-term humidity–frailty association. Annual relative humidity may instead characterize a broader regional climatic moisture environment whose physiological relevance depends on accompanying temperature, acclimatization, housing conditions, access to cooling, and individual adaptive capacity. Accordingly, the estimated indirect effect involving frailty for ambient humidity should be considered biologically plausible but hypothesis-generating rather than confirmation of a causal mechanism. In our data, the model-based mediation analyses suggested that frailty may account for only part of the exposure-associated difference in stroke risk, which is compatible with the presence of parallel pathways involving blood-pressure regulation, vascular dysfunction, thrombogenesis, inflammation, and metabolic disturbance. In CHARLS, the preceding-year exposure assessment, baseline measurement of frailty, and prospective ascertainment of incident stroke provide a more coherent temporal sequence, although frailty was measured only once and residual exposure-mediator and mediator–outcome confounding cannot be excluded. Causal interpretation of the CHARLS estimates additionally depends on the assumptions of consistency, positivity, correct model specification, no unmeasured exposure-outcome, exposure-mediator, or mediator-outcome confounding, and no mediator-outcome confounder affected by the exposure; these assumptions cannot be fully verified in observational data. The hospital-based mediation findings require greater caution because the cross-sectional design cannot establish the temporal ordering of exposure, frailty, and stroke, even though frailty was reconstructed to reflect pre-admission health status. We therefore interpret the hospital-based mediation analysis as an exploratory, hypothesis-generating model-based decomposition and frailty as a potential vulnerability-related pathway rather than a confirmed causal mediator, particularly for ambient humidity (39, 40).

The subgroup analyses are also noteworthy because they point to heterogeneity in vulnerability rather than a uniform exposure effect. In our study, women and participants without work tended to show stronger associations of joint PM_2.5_ and humidity exposure with stroke, particularly for the high PM_2.5_ and high humidity category. This pattern is compatible with recent Chinese evidence from Fang et al., who found stronger PM_2.5_ associated stroke risk among women and among unemployed or agricultural workers in 13,573 middle aged and older adults (30). It is also compatible with the Danish cohort reported by Poulsen et al., where lower socioeconomic position was associated with stronger air-pollution related stroke risk (29). In a cohort of 1,066,752 stroke patients in Sichuan, Cai et al. further reported that higher annual PM_2.5_ was associated with recurrent stroke hospitalization, with an HR of 1.109, 95% CI 1.104 to 1.114, per 10 μg/m^3^, and the association was stronger in females (41). One possible interpretation is that sex-related biological differences, caregiving roles, social stress, occupational insecurity, and fewer material buffers against adverse environments may converge to magnify the impact of environmental exposures. Although alexithymia is a different construct, evidence on its independent risk factors may still be informative because it also concerns vulnerability under chronic stress and limited psychosocial reserve. In a cross-sectional study of 110 patients with axial spondyloarthritis, Cengiz et al. found that female sex was an independent risk factor for alexithymia, with an OR of 22.359, while poorer functional status was also independently associated with alexithymia, with an OR of 1.864 (42). By contrast, Honkalampi et al. studied 137 depressed outpatients and found that male sex, lower education, lower life satisfaction, and more severe depressive symptoms were independently associated with alexithymia (43). Those findings do not map directly onto stroke, but they do suggest that psychosocial vulnerability markers are context dependent, while sex and social adversity repeatedly emerge as modifiers of how individuals absorb and respond to health stressors.

Our findings may have implications for future clinical risk assessment and public-health planning, although they should not be interpreted as evidence that modifying PM_2.5_ or ambient humidity will necessarily reduce stroke risk. The largest effect estimates were observed in the joint high PM_2.5_ and high humidity group, and the model-based mediation analyses suggested that frailty may account for part of these associations. These findings indicate that older adults with frailty and the subgroups identified in the secondary analyses, including women and people without work, may represent potentially susceptible populations; however, these subgroup findings require external confirmation before being used to guide clinical decision-making. In a population-based causal analysis of 502,133 UK Biobank participants, Lin et al. estimated that a hypothetical policy reducing annual PM2.5 exposure by 5% when concentrations exceeded 12 μg/m^3^ would be associated with an absolute 5-year reduction in stroke hospitalization risk of 1.54 per 1,000 people (95% CI 0.73–2.21), illustrating the potential population-health relevance of reducing ambient PM_2.5_ exposure (44). At the individual level, Faridi et al. pooled 17 intervention studies involving approximately 880 participants and reported that portable air cleaners reduced indoor PM_2.5_ by 59.8% and lowered mean systolic blood pressure by 2.35 mmHg, which may be clinically relevant because blood pressure is a major modifiable determinant of stroke (45). In a randomized crossover study of 20 adults with hypertension living in public housing, Wittkopp et al. further observed lower morning home systolic blood pressure during active portable air filtration than during sham filtration, with mean values of 129.6 versus 134.6 mmHg, suggesting that indoor air filtration may be a supportive exposure-reduction strategy for some highly exposed individuals (46). These intervention studies, rather than our observational findings alone, suggest that residential environmental conditions may be considered as contextual information in vascular risk assessment and that improving indoor air quality warrants further investigation; whether such measures reduce incident stroke remains uncertain. For humidity-related risk, heat-health warning systems and targeted communication may offer relevant public-health frameworks, although our annual average relative humidity metric cannot identify short-term hazardous thresholds. Chandra and Lee reviewed 16 heat-health warning systems and concluded that well-designed systems may reduce heat-related morbidity and mortality (47). In a survey of 547 older adults in Queensland, Oberai et al. found that only 43% had heard a heatwave warning and only 49% of those changed their behavior, whereas individuals who perceived themselves to be at heat-related risk were 3.62 times more likely to adopt adaptive measures (48). These findings support further evaluation of combined air-quality and humid-heat communication in primary care, community health management, discharge counseling, and caregiver education, but the present study does not establish that such strategies would prevent stroke. Our mediation findings likewise suggest that frailty may help identify individuals with lower physiological reserve, but they do not demonstrate that routine frailty screening or frailty-directed interventions will modify environment-related stroke risk. In a cohort study of 200 stroke rehabilitation patients aged 65 years or older, Cho et al. reported that greater premorbid frailty was associated with markedly lower odds of discharge home after stroke rehabilitation (OR 0.26, 95% CI 0.17–0.41) and with poorer functional recovery (49), illustrating the broader clinical relevance of frailty assessment in stroke care. Overall, these observations identify potential priorities for future intervention and implementation research, including ambient air-quality improvement, heat-health communication, frailty-informed risk assessment, and additional support for potentially susceptible populations, rather than establishing immediate clinical or policy recommendations.

This study has several strengths. It combined two complementary data sources with different designs, allowing the main associations to be examined in both a nationally based prospective cohort and a clinical hospital-based sample. The CHARLS component provides a more coherent temporal sequence for the assessment of environmental exposure, baseline frailty, and incident stroke, while the hospital-based component provides clinically grounded evidence from patients with documented stroke status. The study also considered PM_2.5_ and humidity jointly rather than separately, evaluated frailty as a prespecified mediator, and explored effect modification in population subgroups that are often underexamined in environmental epidemiology. At the same time, several limitations should be acknowledged. The hospital-based component was cross-sectional, and although frailty deficits were coded to reflect pre-stroke or pre-admission status, the hospital-based mediation analysis was retained only as an exploratory model-based decomposition and cannot establish that frailty temporally or causally mediates the environmental exposure–stroke association. Although the CHARLS analysis had a more coherent temporal structure, its causal interpretation also depends on untestable assumptions, including the absence of unmeasured exposure–mediator, mediator–outcome, and exposure–outcome confounding and the absence of mediator–outcome confounders affected by exposure. Frailty was measured only once at baseline, and changes in frailty during follow-up were not captured. The use of hospitalized non-stroke controls may also introduce selection bias (50, 51). In CHARLS, incident stroke was based on self-reported physician diagnosis rather than adjudicated clinical records, so some outcome misclassification is possible (52). In addition, the exact date of stroke onset was unavailable, and incident stroke was known only to have occurred between the last stroke-free interview and the first positive follow-up interview. Using the first positive interview as the event-time marker in the Cox models may therefore introduce imprecision in event timing. However, the prospective discrete-time analysis, which treated follow-up waves as discrete event intervals, produced estimates that were nearly identical to those from the Cox models, suggesting that the main findings were not materially dependent on this analytical approximation. Exposure assignment was based on annual mean values in the year preceding baseline or the index date and did not capture time-varying exposure, residential mobility, or indoor environmental conditions (53, 54). The ERA5 humidity estimates were available on a 0.25° × 0.25° grid, with a nominal horizontal resolution of approximately 31 km, which was substantially coarser than the 1-km CHAP PM_2.5_ surface. Consequently, participants residing within the same or neighboring ERA5 grid cells could receive identical or highly similar humidity estimates, and local variation related to urban form, elevation, coastal proximity, vegetation, and other microclimatic conditions may have been smoothed. Bilinear interpolation facilitated assignment to residential coordinates but could not recover variability below the native ERA5 resolution. This spatial smoothing may have introduced exposure measurement error and reduced the contrast between participants, although the direction and magnitude of any resulting bias cannot be determined. The ERA5 estimates also represented outdoor ambient conditions and did not capture indoor humidity or individual time–activity patterns. In addition, annual averaging necessarily smooths within-year and seasonal variation and may obscure short periods of extreme humidity or compound humid-heat exposure. Consequently, the present analysis cannot determine whether the observed association was driven by persistent year-round conditions, particular seasons, or shorter episodes in which high humidity co-occurred with high temperature. Dichotomizing PM_2.5_ and humidity by database-specific medians simplified joint exposure modeling but reduced granularity and limited absolute comparability across datasets (55). Residual confounding cannot be excluded despite the additional analyses accounting for baseline multimorbidity, BMI, annual mean ambient temperature, province-level effects, and within-community correlation. In CHARLS, atrial fibrillation was not separately ascertained, and the broader physician-diagnosed heart-disease item could not fully distinguish specific cardiac conditions. In addition, annual mean temperature was modeled as a continuous covariate and may not fully capture nonlinear, seasonal, or short-term thermal effects. Province-level fixed effects controlled for broad regional differences, and community-clustered variance estimates accounted for within-community statistical dependence, but residual finer-scale spatial confounding may remain. The spatial sensitivity analysis was limited to CHARLS because consistent residential-community identifiers were unavailable in the hospital dataset and most hospital-based participants resided in Fujian Province, limiting the geographic variation required for stable province-level estimation. Residual confounding by diet, medication use, healthcare access, indoor environmental conditions, and finer-scale neighborhood characteristics therefore remains possible, and the complete-case approach may also have introduced selection effects (56). Stroke subtype specific analyses were not performed, and future work with repeated exposure assessment, validated stroke subtyping, and prospectively measured frailty would help clarify these relationships.

## 5 Conclusion

In this study integrating a population-based prospective cohort and a single-center hospital-based sample, higher PM_2.5_ exposure and higher ambient relative humidity were each associated with less favorable stroke outcomes, and their joint presence showed a consistent pattern of greater stroke risk, with the largest effect estimates observed in the high PM_2.5_ and high humidity category. In CHARLS, model-based mediation analysis suggested a potential partial indirect pathway involving frailty, indicating that differences in frailty burden may account for part of the observed associations alongside other pathways not captured by frailty. The corresponding hospital-based mediation findings were exploratory because the cross-sectional design could not establish the temporal ordering of environmental exposure, frailty, and stroke. Exploratory subgroup analyses also suggested possible heterogeneity by sex and employment status, with generally stronger associations among women and participants without work, although these findings require confirmation in independent populations. Overall, the findings suggest that the joint environmental context of PM_2.5_ and ambient humidity may be relevant to future research on stroke-risk assessment and that frailty may represent a potential vulnerability-related pathway. Further prospective studies incorporating repeated exposure and frailty assessments, more detailed control of thermal and spatial factors, and validated stroke subtyping are needed to strengthen causal inference before these observations can be translated into clinical or public-health recommendations.

## Data Availability

The original contributions presented in the study are included in the article/Supplementary material, further inquiries can be directed to the corresponding author.

## Footnotes

1
https://weijing-rs.github.io/product.html
