# Supplementary material for: When air gets heavy: joint PM2.5 and ambient humidity exposure, frailty, and stroke
Source: Front Public Health. 2026 Sep 9;14:1883147. doi: 10.3389/fpubh.2026.1883147 (PMC13597862; doi:10.3389/fpubh.2026.1883147)
Supplement: Supplementary file 1 [file Data_Sheet_1.DOCX]

**Appendix 1. Detailed components and coding rules of the frailty index**

**A1.1 Overview of frailty index construction**

Frailty was operationalized in both data sources using a harmonized 40-item deficit-accumulation frailty index (FI) constructed according to standard deficit-accumulation principles. Each deficit was coded on a scale from 0 to 1, with higher values indicating worse health status or greater impairment. The FI for each participant was calculated as the sum of all non-missing deficit scores divided by the number of deficits assessed. The resulting FI therefore ranged from 0 to 1, with higher values indicating greater frailty burden.

To preserve comparability between the CHARLS cohort and the single-center hospital-based study, the analytic FI in the present study was based on 40 deficits. The initial CHARLS candidate pool contained 42 items. Two items, calling and running or jogging about 1 km, were not included in the final harmonized FI because equivalent pre-admission information was less consistently available in the hospital-based records and these items showed lower cross-source comparability. Thus, the final FI used in the present study consisted of 40 items spanning activities of daily living, instrumental activities of daily living, physical functioning, chronic diseases, psychological status, self-rated health, and related indicators of physiological reserve.

Participants were required to have at least 30 non-missing deficits to be included in frailty analyses. For descriptive analyses, frailty was additionally classified using the conventional cut-off FI ≥ 0.25.

In the single-center hospital-based study, all frailty deficits were coded to reflect the participant’s usual pre-stroke or pre-admission health status, using a predefined source hierarchy that included the admission record, past medical history, pre-admission functional documentation, routine nursing assessment, and patient or family report. Acute stroke-related manifestations and in-hospital complications were excluded from FI construction.

**A1.2 Source hierarchy and general coding rules in the hospital-based study**

For the hospital-based dataset, deficit coding followed the hierarchy below:

1. Past medical history and discharge summary for chronic diseases and previously documented functional limitations
2. Admission record and nursing intake assessment for pre-admission activities of daily living, mobility, continence, and usual functional status
3. Patient or family report for pre-admission psychological symptoms, sleep quality, and self-rated health when explicitly documented
4. If a deficit could not be reasonably assigned to the participant’s pre-admission status, the item was coded as missing rather than inferred from the acute hospitalization

Additional rules were applied as follows:

- Repeated mentions of the same condition were counted once only.
- The diagnosis of the index stroke was excluded by design.
- Acute complications arising during hospitalization were not counted as chronic deficits.
- For ordinal functional items, coding reflected the participant’s usual level before the index event, not the level observed after acute stroke onset.

**A1.3 Deficit items included in the harmonized 40-item frailty index**

**Table A1.1.** Components and coding rules of the harmonized 40-item frailty index

| **Domain** | **No.** | **Deficit item** | **Coding rule** |
| --- | --- | --- | --- |
| Activities of daily living | 1 | Bathing | No difficulty = 0; some difficulty = 0.33; needs help = 0.67; unable/dependent = 1 |
|  | 2 | Dressing | No difficulty = 0; some difficulty = 0.33; needs help = 0.67; unable/dependent = 1 |
|  | 3 | Using the toilet | No difficulty = 0; some difficulty = 0.33; needs help = 0.67; unable/dependent = 1 |
|  | 4 | Transferring | No difficulty = 0; some difficulty = 0.33; needs help = 0.67; unable/dependent = 1 |
|  | 5 | Continence | No difficulty = 0; some difficulty = 0.33; needs help = 0.67; unable/dependent = 1 |
|  | 6 | Eating | No difficulty = 0; some difficulty = 0.33; needs help = 0.67; unable/dependent = 1 |
| Instrumental activities of daily living | 7 | Cooking | No difficulty = 0; some difficulty = 0.33; needs help = 0.67; unable/dependent = 1 |
|  | 8 | Shopping | No difficulty = 0; some difficulty = 0.33; needs help = 0.67; unable/dependent = 1 |
|  | 9 | Doing household chores | No difficulty = 0; some difficulty = 0.33; needs help = 0.67; unable/dependent = 1 |
|  | 10 | Taking medicine | No difficulty = 0; some difficulty = 0.33; needs help = 0.67; unable/dependent = 1 |
|  | 11 | Managing money | No difficulty = 0; some difficulty = 0.33; needs help = 0.67; unable/dependent = 1 |
| Physical functional limitations | 12 | Lifting a weight of 5 kg | No difficulty = 0; some difficulty = 0.33; great difficulty = 0.67; unable = 1 |
|  | 13 | Walking 1 km | No difficulty = 0; some difficulty = 0.33; great difficulty = 0.67; unable = 1 |
|  | 14 | Walking 100 m | No difficulty = 0; some difficulty = 0.33; great difficulty = 0.67; unable = 1 |
|  | 15 | Stooping, kneeling, or crouching | No difficulty = 0; some difficulty = 0.33; great difficulty = 0.67; unable = 1 |
|  | 16 | Standing up from sitting | No difficulty = 0; some difficulty = 0.33; great difficulty = 0.67; unable = 1 |
|  | 17 | Picking up a coin from a table | No difficulty = 0; some difficulty = 0.33; great difficulty = 0.67; unable = 1 |
|  | 18 | Reaching or extending arms above shoulder level | No difficulty = 0; some difficulty = 0.33; great difficulty = 0.67; unable = 1 |
|  | 19 | Climbing several flights of stairs without resting | No difficulty = 0; some difficulty = 0.33; great difficulty = 0.67; unable = 1 |
| Chronic diseases | 20 | Chronic lung disease (including chronic bronchitis or emphysema) | No = 0; Yes = 1 |
|  | 21 | Asthma | No = 0; Yes = 1 |
|  | 22 | Cancer or malignant tumor | No = 0; Yes = 1 |
|  | 23 | Arthritis or rheumatism | No = 0; Yes = 1 |
|  | 24 | Gastric or duodenal ulcer / chronic digestive disease | No = 0; Yes = 1 |
|  | 25 | Kidney disease | No = 0; Yes = 1 |
|  | 26 | Hypertension | No = 0; Yes = 1 |
|  | 27 | Dyslipidemia | No = 0; Yes = 1 |
|  | 28 | Diabetes / abnormal blood glucose | No = 0; Yes = 1 |
|  | 29 | Heart disease (including myocardial infarction, coronary artery disease, angina, congestive heart failure, or other cardiac conditions) | No = 0; Yes = 1 |
|  | 30 | Emotional, nervous, or psychiatric problems | No = 0; Yes = 1 |
|  | 31 | Liver disease | No = 0; Yes = 1 |
|  | 32 | Memory-related disease (including dementia, brain atrophy, or Parkinsonism) | No = 0; Yes = 1 |
| Mental health and related symptoms | 33 | Feeling depressed | Rarely or never = 0; sometimes = 0.33; often = 0.67; most or all of the time = 1 |
|  | 34 | Feeling fearful | Rarely or never = 0; sometimes = 0.33; often = 0.67; most or all of the time = 1 |
|  | 35 | Feeling unable to continue living | Rarely or never = 0; sometimes = 0.33; often = 0.67; most or all of the time = 1 |
|  | 36 | Feeling happy | Most or all of the time = 0; often = 0.33; sometimes = 0.67; rarely or never = 1 |
|  | 37 | Feeling hopeful about the future | Most or all of the time = 0; often = 0.33; sometimes = 0.67; rarely or never = 1 |
|  | 38 | Poor sleep quality | Rarely or never = 0; sometimes = 0.33; often = 0.67; most or all of the time = 1 |
|  | 39 | Feeling that doing things is very effortful | Rarely or never = 0; sometimes = 0.33; often = 0.67; most or all of the time = 1 |
| Self-rated health | 40 | Self-rated health | Very good = 0; good = 0.25; average = 0.50; poor = 0.75; very poor = 1 |

Poor sleep quality in the frailty index was distinct from the sleep-duration covariate used in the regression models. Sleep duration was based on reported sleep hours and categorized as <6 h, 6–8 h, or >8 h, whereas the frailty deficit reflected the reported frequency of poor sleep quality. Sleep duration itself was not included as a frailty-index item. Therefore, the same sleep variable was not counted twice, and no duplicated statistical weight was assigned.

**A1.4 Candidate items not included in the final harmonized FI**

Two items from the original broader CHARLS candidate list were not included in the final harmonized 40-item FI:

**Table A1.2.** Candidate deficit items excluded from the final harmonized FI

| **Candidate item** | **Reason for exclusion** |
| --- | --- |
| Calling | Lower comparability across data sources; pre-admission documentation less consistently available in the hospital-based study |
| Running or jogging about 1 km | Lower comparability across data sources; less consistently documented as usual pre-admission function in the hospital-based study |

These exclusions were made to preserve cross-dataset harmonization and to ensure that the FI reflected comparable constructs in both the community-based and hospital-based samples.

**A1.5 Availability of frailty deficit data in the analytic samples**

After exclusion of participants with missing frailty data, all included participants met the prespecified requirement of having at least 30 assessable deficits.

**Table A1.3.** Availability of frailty deficit data and summary FI distribution in the analytic samples

| **Characteristic** | **CHARLS** | **Single-center hospital-based study** |
| --- | --- | --- |
| Analytic sample size | 17,197 | 782 |
| Mean number of non-missing deficits assessed, mean (SD) | 39.2 (1.1) | 36.8 (2.3) |
| Median number of non-missing deficits assessed (IQR) | 40 (39–40) | 37 (35–39) |
| Participants with ≥30 non-missing deficits, n (%) | 17,197 (100.0) | 782 (100.0) |
| Frailty index, mean (SD) | 0.13 (0.09) | 0.19 (0.11) |
| Frail (FI ≥ 0.25), n (%) | 1,703 (9.9) | 229 (29.3) |

These summary statistics were consistent with the distributions reported in the main descriptive table and supported adequate coverage of FI items in both data sources.

**A1.6 Interpretation and rationale**

The harmonized 40-item FI was designed to capture multidimensional vulnerability across both data sources while maintaining consistency in conceptual scope and coding rules. The use of a deficit-accumulation approach allowed functional impairment, chronic disease burden, psychological symptoms, sleep, and self-rated health to be integrated into a single continuous measure of frailty. The requirement of at least 30 non-missing deficits ensured adequate measurement reliability, and the additional frailty classification using FI ≥ 0.25 facilitated descriptive comparisons across groups.

In the hospital-based study, particular care was taken to ensure that FI coding reflected the participant’s usual pre-stroke or pre-admission state rather than acute stroke-related impairment. This was achieved through a predefined source hierarchy, exclusion of acute manifestations and in-hospital complications, and conservative coding of uncertain items as missing rather than positive deficits. Taken together, these procedures support the use of the FI as a prespecified mediator in the primary and supplementary analyses.

Although chronic diseases formed one domain of the frailty index, the index was not a comorbidity score. Each fully expressed deficit contributed the same maximum amount to the overall index—1/40 when all 40 deficits were assessed—regardless of whether the deficit represented a chronic disease, functional limitation, psychological symptom, or self-rated-health impairment.

**Appendix 2. Detailed results of model diagnostic assessments**

**A2.1 Diagnostic assessment of the Cox proportional hazards models in CHARLS**

The proportional hazards assumption for the fully adjusted Cox models in CHARLS was evaluated using Schoenfeld residual-based tests. Neither the exposure variables nor the covariates showed evidence of violation of the proportional hazards assumption. The global test was not statistically significant, supporting the adequacy of the Cox modeling framework for the primary analyses.

**Table A2.1. Schoenfeld residual-based tests for the fully adjusted Cox model in CHARLS**

| **Variable** | **Chi-square** | **df** | **P value** |
| --- | --- | --- | --- |
| PM_2.5_ group | 1.84 | 1 | 0.175 |
| Relative humidity group | 1.27 | 1 | 0.260 |
| Age | 2.09 | 1 | 0.148 |
| Sex | 0.91 | 1 | 0.340 |
| Marital status | 0.66 | 1 | 0.417 |
| Residence | 1.12 | 1 | 0.289 |
| Smoking status | 2.41 | 2 | 0.300 |
| Alcohol drinking status | 1.86 | 2 | 0.394 |
| Income group | 0.58 | 1 | 0.448 |
| Sleep duration group | 2.67 | 2 | 0.263 |
| Number of living children | 3.19 | 3 | 0.364 |
| Employment status | 2.94 | 2 | 0.230 |
| **Global test** | **14.31** | **18** | **0.709** |

**Interpretation:** The global Schoenfeld residual test jointly evaluated all 18 regression coefficients represented by the covariates listed above, including the indicator coefficients for multicategory variables. The global test was not statistically significant (χ² = 14.31, df = 18, P = 0.709), and none of the term-specific tests was statistically significant (all P > 0.05), indicating no statistical evidence of departure from the proportional hazards assumption in the primary CHARLS Cox model. The P values shown in this table are diagnostic P values for the proportional hazards assumption and should not be interpreted as tests of the exposure–stroke associations.

**A2.2 Diagnostic assessment of the multivariable logistic regression models in the single-center hospital-based study**

For the hospital-based logistic regression analyses, model adequacy was assessed by examining goodness of fit, calibration, multicollinearity, influential observations, and discrimination. The fully adjusted model showed acceptable fit in the Hosmer–Lemeshow test, satisfactory calibration of predicted probabilities, no evidence of problematic multicollinearity, and no influential observations that materially altered the regression coefficients. Model discrimination was acceptable.

**Table A2.2. Goodness-of-fit and discrimination statistics for the fully adjusted logistic regression model in the hospital-based study**

| **Diagnostic measure** | **Value** |
| --- | --- |
| Hosmer–Lemeshow chi-square | 7.84 |
| Degrees of freedom | 8 |
| Hosmer–Lemeshow P value | 0.449 |
| Calibration intercept | 0.03 |
| Calibration slope | 0.97 |
| Area under the ROC curve (AUC) | 0.761 |
| 95% CI for AUC | 0.719–0.803 |

**Interpretation:** The non-significant Hosmer–Lemeshow test and calibration slope close to 1.0 indicated acceptable agreement between observed and predicted stroke probabilities. The AUC of 0.761 suggested acceptable discriminatory performance of the fully adjusted model.

**A2.3 Multicollinearity assessment in the hospital-based logistic regression model**

Variance inflation factors were calculated for all variables included in the fully adjusted logistic regression model. All VIF values were low and well below conventional thresholds for problematic multicollinearity.

**Table A2.3. Variance inflation factors for covariates in the fully adjusted logistic regression model**

| **Variable** | **VIF** |
| --- | --- |
| PM_2.5_ group | 1.42 |
| Relative humidity group | 1.37 |
| Age | 1.29 |
| Sex | 1.18 |
| Marital status | 1.11 |
| Residence | 1.24 |
| Smoking status | 1.53 |
| Alcohol drinking status | 1.47 |
| Income group | 1.21 |
| Sleep duration group | 1.34 |
| Number of living children | 1.19 |
| Employment status | 1.58 |

| **Summary statistic** | **Value** |
| --- | --- |
| Mean VIF | 1.33 |
| Maximum VIF | 1.58 |

**Interpretation:** All VIF values were below 2.0, indicating no evidence of problematic multicollinearity among the predictors in the primary hospital-based logistic regression model.

**A2.4 Assessment of influential observations in the hospital-based logistic regression model**

Influential observations were assessed using leverage values, standardized Pearson residuals, deviance residuals, Cook’s distance, and DFBETA diagnostics. A small number of observations showed moderately elevated leverage or residual values, but none met conventional criteria for undue influence on the overall model estimates.

**Table A2.4. Influence diagnostics for the fully adjusted logistic regression model**

| **Diagnostic measure** | **Value** |
| --- | --- |
| Number of observations | 782 |
| Mean leverage | 0.015 |
| Maximum leverage | 0.046 |
| Observations with leverage > 2 × mean leverage | 21 |
| Observations with leverage > 3 × mean leverage | 6 |
| Standardized Pearson residual range | -2.41 to 2.67 |
| Deviance residual range | -2.29 to 2.54 |
| Maximum Cook’s distance | 0.041 |
| Observations with Cook’s distance > 0.50 | 0 |
| Observations with Cook’s distance > 1.00 | 0 |
| Maximum absolute DFBETA | 0.23 |

**Interpretation:** Although a small number of observations showed somewhat higher leverage, none had Cook’s distance or DFBETA values suggesting undue influence on the fitted coefficients. Re-estimation after temporary removal of the six highest-leverage observations changed the principal exposure effect estimates by less than 5%, indicating that the main findings were not driven by a small number of influential cases.

**Overall interpretation of model diagnostics**

Taken together, the diagnostic assessments supported the adequacy of the primary modeling strategy in both data sources. In CHARLS, the Schoenfeld residual tests indicated no meaningful violation of the proportional hazards assumption. In the hospital-based study, the logistic regression models showed acceptable fit, calibration, and discrimination, with no problematic multicollinearity or influential observations. These results support the validity of the primary regression-based estimates reported in the main analyses.

**Appendix 3. Detailed components and coding rules of the comorbidity index**

**A3.1 Purpose and general approach**

To examine whether the observed associations of PM_2.5_ exposure and ambient relative humidity with stroke were materially altered after accounting for baseline chronic disease burden, a comorbidity index was constructed separately in the CHARLS cohort and in the single-center hospital-based study. In both data sources, the index was defined as an unweighted count of pre-existing chronic diseases, with higher values indicating greater background multimorbidity burden. The purpose of this index was to capture baseline chronic disease load rather than acute illness severity or the stroke outcome itself.

Across both datasets, the diagnosis of stroke was excluded by design from the comorbidity index. Acute complications arising during the index hospitalization were also excluded in the hospital-based study. Unless otherwise specified, each condition contributed one point to the index if present and zero points if absent. The final score was calculated as the sum of all eligible conditions.

**A3.2 Construction of the comorbidity index in CHARLS**

In the CHARLS cohort, the comorbidity index was derived from baseline self-reported physician-diagnosed chronic disease indicators available in the dataset. Participants with prevalent stroke at baseline had already been excluded from the analytic sample and therefore stroke was not included as an index component. Conditions were coded as present if the participant reported that a doctor had ever diagnosed the disease at or before the baseline interview.

The following chronic conditions were included in the CHARLS comorbidity index:

1. Hypertension
2. Diabetes or high blood sugar
3. Dyslipidemia
4. Heart disease
5. Chronic lung disease
6. Liver disease
7. Kidney disease
8. Stomach or other digestive disease
9. Arthritis or rheumatism
10. Asthma
11. Memory-related disease
12. Psychiatric or emotional disorder
13. Malignant tumor or cancer

Each condition was coded as 1 if present and 0 if absent. The CHARLS comorbidity index therefore ranged from 0 to 13.

**Table A3.1. Components of the CHARLS comorbidity index and coding rules**

| **Component** | **Coding rule** |
| --- | --- |
| Hypertension | 1 if physician-diagnosed hypertension was reported at baseline; otherwise 0 |
| Diabetes or high blood sugar | 1 if physician-diagnosed diabetes or high blood sugar was reported at baseline; otherwise 0 |
| Dyslipidemia | 1 if physician-diagnosed dyslipidemia was reported at baseline; otherwise 0 |
| Heart disease | 1 if physician-diagnosed heart disease was reported at baseline; otherwise 0 |
| Chronic lung disease | 1 if physician-diagnosed chronic lung disease was reported at baseline; otherwise 0 |
| Liver disease | 1 if physician-diagnosed liver disease was reported at baseline; otherwise 0 |
| Kidney disease | 1 if physician-diagnosed kidney disease was reported at baseline; otherwise 0 |
| Digestive disease | 1 if physician-diagnosed stomach or other digestive disease was reported at baseline; otherwise 0 |
| Arthritis or rheumatism | 1 if physician-diagnosed arthritis or rheumatism was reported at baseline; otherwise 0 |
| Asthma | 1 if physician-diagnosed asthma was reported at baseline; otherwise 0 |
| Memory-related disease | 1 if physician-diagnosed memory-related disease was reported at baseline; otherwise 0 |
| Psychiatric disorder | 1 if physician-diagnosed psychiatric or emotional disorder was reported at baseline; otherwise 0 |
| Malignant tumor | 1 if physician-diagnosed cancer or malignant tumor was reported at baseline; otherwise 0 |

**Distribution of the CHARLS comorbidity index**

In the analytic CHARLS sample (n = 17,197), the mean comorbidity index was 1.13 (SD 1.18) and the median was 1 (IQR 0–2). Among participants who remained stroke-free during follow-up, the mean index was 1.07 (SD 1.14), compared with 1.72 (SD 1.34) among those who developed incident stroke.

**Table A3.2.** Distribution of the CHARLS comorbidity index by incident stroke status

| **Comorbidity index category** | **Total, n (%)** | **No incident stroke, n (%)** | **Incident stroke, n (%)** |
| --- | --- | --- | --- |
| 0 | 6,281 (36.5) | 5,915 (37.6) | 366 (24.6) |
| 1 | 5,344 (31.1) | 4,936 (31.4) | 408 (27.5) |
| 2 | 3,224 (18.8) | 2,881 (18.3) | 343 (23.1) |
| ≥3 | 2,348 (13.7) | 1,980 (12.6) | 368 (24.8) |

These distributions indicate a higher baseline multimorbidity burden among participants who later developed stroke, supporting the use of the index as an additional adjustment variable in sensitivity analyses.

**A3.3 Construction of the comorbidity index in the single-center hospital-based study**

In the single-center hospital-based study, the comorbidity index was derived from pre-existing chronic conditions documented in the admission record, past medical history section, discharge summary, and inpatient problem list. To preserve the intended meaning of background multimorbidity burden, only conditions explicitly documented as present before or at the time of admission were counted. Conditions judged to represent acute complications of the current hospitalization were not counted. The diagnosis of the index stroke itself was excluded by design.

The following chronic conditions were eligible for inclusion:

1. Hypertension
2. Diabetes mellitus
3. Dyslipidemia
4. Coronary heart disease
5. Heart failure
6. Atrial fibrillation or other clinically documented arrhythmias
7. Chronic kidney disease
8. Chronic liver disease
9. Chronic lung disease
10. Malignant tumor
11. Chronic digestive disease
12. Arthritis or rheumatic disease
13. Asthma
14. Psychiatric disorder
15. Memory-related disease or Parkinsonism

Each condition was coded as 1 if present and 0 if absent. The hospital-based comorbidity index therefore ranged from 0 to 15.

**Table A3.3.** Components of the hospital-based comorbidity index and coding rules

| **Component** | **Coding rule** |
| --- | --- |
| Hypertension | 1 if documented as pre-existing before or at admission; otherwise 0 |
| Diabetes mellitus | 1 if documented as pre-existing before or at admission; otherwise 0 |
| Dyslipidemia | 1 if documented as pre-existing before or at admission; otherwise 0 |
| Coronary heart disease | 1 if documented as pre-existing before or at admission; otherwise 0 |
| Heart failure | 1 if documented as pre-existing before or at admission; otherwise 0 |
| Atrial fibrillation or other arrhythmia | 1 if documented as pre-existing before or at admission; otherwise 0 |
| Chronic kidney disease | 1 if documented as pre-existing before or at admission; otherwise 0 |
| Chronic liver disease | 1 if documented as pre-existing before or at admission; otherwise 0 |
| Chronic lung disease | 1 if documented as pre-existing before or at admission; otherwise 0 |
| Malignant tumor | 1 if documented as pre-existing before or at admission; otherwise 0 |
| Chronic digestive disease | 1 if documented as pre-existing before or at admission; otherwise 0 |
| Arthritis or rheumatic disease | 1 if documented as pre-existing before or at admission; otherwise 0 |
| Asthma | 1 if documented as pre-existing before or at admission; otherwise 0 |
| Psychiatric disorder | 1 if documented as pre-existing before or at admission; otherwise 0 |
| Memory-related disease or Parkinsonism | 1 if documented as pre-existing before or at admission; otherwise 0 |

Additional coding rules for the hospital-based study

1. The index stroke diagnosis was excluded in all stroke cases.
2. Acute conditions arising during hospitalization, such as aspiration pneumonia, acute kidney injury, stress hyperglycemia, delirium, or other new complications, were not counted.
3. If a chronic condition was mentioned only as a possible differential diagnosis and not confirmed in the final clinical documentation, it was coded as absent.
4. Repeated mentions of the same disease across different parts of the record were counted only once.
5. Where documentation used synonymous diagnostic terms, coding was harmonized to a single disease category according to the prespecified rules.

**Distribution of the hospital-based comorbidity index**

In the hospital-based analytic sample (n = 782), the mean comorbidity index was 2.58 (SD 1.74) and the median was 2 (IQR 1–4). The mean index was 2.39 (SD 1.66) in non-stroke controls and 3.37 (SD 1.82) in stroke patients.

**Table A3.4. Distribution of the hospital-based comorbidity index by stroke status**

| **Comorbidity index category** | **Total, n (%)** | **Non-stroke, n (%)** | **Stroke, n (%)** |
| --- | --- | --- | --- |
| 0 | 96 (12.3) | 89 (14.0) | 7 (4.7) |
| 1 | 183 (23.4) | 161 (25.4) | 22 (14.9) |
| 2 | 189 (24.2) | 158 (24.9) | 31 (20.9) |
| ≥3 | 314 (40.2) | 226 (35.6) | 88 (59.5) |

These distributions indicate a greater background multimorbidity burden among stroke patients than among hospitalized non-stroke controls.

**A3.4 Use of the comorbidity index in sensitivity analyses**

The comorbidity index was included only as an additional covariate in a separate sensitivity analysis to evaluate whether the main associations of PM_2.5_ exposure and ambient relative humidity with stroke were materially altered after accounting for baseline chronic disease burden. The frailty index and the comorbidity index served distinct analytical purposes. The frailty index was treated as the prespecified mediator in the mediation analyses, whereas the comorbidity index was used to assess the robustness of the exposure–stroke associations to additional control for background multimorbidity. The two indices were not entered simultaneously in the same exposure–stroke model. Therefore, although some chronic disease indicators contributed to both derived measures, these diseases were not counted twice within a single regression model and did not receive duplicated statistical weight.

Frailty and comorbidity were retained as separate constructs because they represent different dimensions of health. The comorbidity index was an unweighted count of diagnosed chronic diseases and therefore primarily reflected multimorbidity burden. By contrast, the 40-item frailty index was a multidimensional deficit-accumulation measure that incorporated activities of daily living, instrumental activities of daily living, physical functioning, chronic diseases, psychological symptoms, sleep quality, and self-rated health. Frailty therefore captured broader loss of physiological and functional reserve that could not be represented by a disease count alone. Because the study specifically aimed to evaluate frailty as a potential vulnerability-related pathway between environmental exposure and stroke, replacing the frailty index with the comorbidity index would have addressed a different scientific question.

The comorbidity index was not included in the primary exposure–stroke models because several of its disease components were also included in the prespecified frailty mediator and may partly lie on pathways linking long-term environmental exposure with stroke. Routine adjustment for these conditions in the primary models could therefore condition on components of the proposed intermediate pathway, attenuate part of the total exposure association, and introduce overadjustment. Nevertheless, given the established clinical importance of vascular and other chronic diseases for stroke, the comorbidity index was included in a dedicated sensitivity analysis to determine whether the principal findings could be explained by differences in pre-existing disease burden.

After additional adjustment for the comorbidity index, the effect estimates were modestly attenuated in both data sources, but the principal associations remained statistically significant and the overall pattern was unchanged. In particular, the combined high PM_2.5_ and high humidity category continued to show the largest association with stroke. These findings indicate that the observed associations were not explained solely by differences in background multimorbidity burden and support the robustness of the primary conclusions.

**A3.5 Overall interpretation**

The comorbidity index was designed to provide a pragmatic measure of baseline multimorbidity burden while preserving conceptual distinction from both the frailty index and the stroke outcome. Across both the community-based cohort and the hospital-based sample, higher comorbidity burden was more common among participants with stroke, but adjustment for this index did not materially alter the primary exposure–stroke associations. This supports the robustness of the main findings and suggests that the observed associations of PM_2.5_ and ambient humidity with stroke were not driven solely by differences in pre-existing chronic disease burden.

**Appendix 4.** Subgroup analysis for the association of joint PM_2.5_ and humidity exposure with stroke in CHARLS and the single-center hospital-based study

| **Variables** | **HR/OR (95% CI)** | ***P*-value** | ***P* for interaction** |
| --- | --- | --- | --- |
| **CHARLS** |  |  |  |
| **Sex** |  |  | 0.021 |
| **Female** |  |  |  |
| High PM_2.5_ and low humidity vs. low PM_2.5_ and low humidity | 1.304 (1.043–1.631) | 0.028 |  |
| Low PM_2.5_ and high humidity vs. low PM_2.5_ and low humidity | 1.258 (1.006–1.572) | 0.044 |  |
| High PM_2.5_ and high humidity vs. low PM_2.5_ and low humidity | 1.548 (1.234–1.942) | <0.001 |  |
| **Male** |  |  |  |
| High PM_2.5_ and low humidity vs. low PM_2.5_ and low humidity | 1.161 (0.930–1.449) | 0.186 |  |
| Low PM_2.5_ and high humidity vs. low PM_2.5_ and low humidity | 1.139 (0.910–1.425) | 0.255 |  |
| High PM_2.5_ and high humidity vs. low PM_2.5_ and low humidity | 1.247 (1.001–1.554) | 0.049 |  |
|  |  |  |  |
| **Employment status** | |  | <0.001 |
| **No work** |  |  |  |
| High PM_2.5_ and low humidity vs. low PM_2.5_ and low humidity | 1.401 (1.097–1.790) | 0.007 |  |
| Low PM_2.5_ and high humidity vs. low PM_2.5_ and low humidity | 1.336 (1.042–1.713) | 0.023 |  |
| High PM_2.5_ and high humidity vs. low PM_2.5_ and low humidity | 1.664 (1.294–2.140) | <0.001 |  |
| **Agricultural work** |  |  |  |
| High PM_2.5_ and low humidity vs. low PM_2.5_ and low humidity | 1.220 (0.988–1.507) | 0.064 |  |
| Low PM_2.5_ and high humidity vs. low PM_2.5_ and low humidity | 1.176 (0.949–1.457) | 0.138 |  |
| High PM_2.5_ and high humidity vs. low PM_2.5_ and low humidity | 1.347 (1.085–1.671) | 0.007 |  |
| **Non-agricultural work** |  |  |  |
| High PM_2.5_ and low humidity vs. low PM_2.5_ and low humidity | 1.097 (0.822–1.464) | 0.527 |  |
| Low PM_2.5_ and high humidity vs. low PM_2.5_ and low humidity | 1.112 (0.833–1.483) | 0.473 |  |
| High PM_2.5_ and high humidity vs. low PM_2.5_ and low humidity | 1.201 (0.907–1.590) | 0.196 |  |
|  |  |  |  |
| **Single-center hospital-based study** | | | |
| **Sex** |  |  | <0.001 |
| **Female** |  |  |  |
| High PM_2.5_ and low humidity vs. low PM_2.5_ and low humidity | 1.938 (0.920–4.082) | 0.081 |  |
| Low PM_2.5_ and high humidity vs. low PM_2.5_ and low humidity | 2.067 (0.986–4.335) | 0.054 |  |
| High PM_2.5_ and high humidity vs. low PM_2.5_ and low humidity | 3.012 (1.368–6.631) | 0.006 |  |
| **Male** |  |  |  |
| High PM_2.5_ and low humidity vs. low PM_2.5_ and low humidity | 1.523 (0.739–3.141) | 0.254 |  |
| Low PM_2.5_ and high humidity vs. low PM_2.5_ and low humidity | 1.588 (0.771–3.272) | 0.211 |  |
| High PM_2.5_ and high humidity vs. low PM_2.5_ and low humidity | 2.203 (1.061–4.577) | 0.034 |  |
|  |  |  |  |
| **Employment status** | |  | 0.003 |
| **No work** |  |  |  |
| High PM_2.5_ and low humidity vs. low PM_2.5_ and low humidity | 2.181 (1.042–4.565) | 0.039 |  |
| No work: Low PM_2.5_ and high humidity vs. low PM_2.5_ and low humidity | 2.274 (1.088–4.752) | 0.029 |  |
| No work: High PM_2.5_ and high humidity vs. low PM_2.5_ and low humidity | 3.284 (1.575–6.845) | 0.002 |  |
| **Agricultural work** |  |  |  |
| High PM_2.5_ and low humidity vs. low PM_2.5_ and low humidity | 1.452 (0.578–3.648) | 0.428 |  |
| Low PM_2.5_ and high humidity vs. low PM_2.5_ and low humidity | 1.531 (0.608–3.855) | 0.365 |  |
| High PM_2.5_ and high humidity vs. low PM_2.5_ and low humidity | 2.118 (0.902–4.974) | 0.084 |  |
| **Non-agricultural work** |  |  |  |
| High PM_2.5_ and low humidity vs. low PM_2.5_ and low humidity | 1.336 (0.494–3.614) | 0.57 |  |
| Low PM_2.5_ and high humidity vs. low PM_2.5_ and low humidity | 1.401 (0.519–3.780) | 0.506 |  |
| High PM_2.5_ and high humidity vs. low PM_2.5_ and low humidity | 1.856 (0.747–4.609) | 0.183 |  |

All models were adjusted for age, sex, marital status, residence, smoking status, alcohol drinking status, income group, sleep duration group, number of living children, and employment status, except for the variable used for stratification. In CHARLS, estimates are presented as HRs from fully adjusted Cox proportional hazards models; in the single-center hospital-based study, estimates are presented as ORs from fully adjusted multivariable logistic regression models. The reference group in all subgroup analyses was the low PM_2.5_ and low humidity category. The reported *P* values for interaction were obtained from global tests of all interaction terms between the four-category joint exposure variable and the corresponding modifier. These P values assess heterogeneity of the overall joint exposure association across strata and should not be interpreted as *P* values for individual stratum-specific HRs or ORs. Only modifiers with statistically significant global interaction in both datasets are presented.

**Explanation:** Interaction testing indicated that sex and employment status were the only effect modifiers that showed statistically significant interaction with the joint PM_2.5_–humidity exposure in both datasets; therefore, stratified analyses were performed for these two variables. Across both CHARLS and the single-center hospital-based study, the overall pattern was similar: compared with the reference category of low PM_2.5_ and low humidity, the combined exposure categories tended to be associated with higher stroke risk, with the largest effect estimates generally observed for high PM_2.5_ and high humidity. In addition, women and participants without work tended to show stronger associations than men and those engaged in agricultural or non-agricultural work, respectively.

In CHARLS, sex significantly modified the association between the joint exposure and incident stroke (*P* for interaction = 0.021). Among women, all three non-reference categories were associated with higher incident stroke risk, including high PM_2.5_ and low humidity (HR 1.304, 95% CI 1.043–1.631), low PM_2.5_ and high humidity (HR 1.258, 95% CI 1.006–1.572), and high PM_2.5_ and high humidity (HR 1.548, 95% CI 1.234–1.942), with the largest elevation observed for the combined high PM_2.5_–high humidity exposure. Among men, effect estimates were directionally similar but generally smaller and less precise; neither high PM_2.5_ and low humidity (HR 1.161, 95% CI 0.930–1.449) nor low PM_2.5_ and high humidity (HR 1.139, 95% CI 0.910–1.425) was statistically significant, whereas high PM_2.5_ and high humidity remained positively associated with incident stroke (HR 1.247, 95% CI 1.001–1.554).

Employment status also significantly modified the association in CHARLS (*P* for interaction <0.001). Among participants with no work, all three joint exposure categories were associated with higher incident stroke risk, including high PM_2.5_ and low humidity (HR 1.401, 95% CI 1.097–1.790), low PM_2.5_ and high humidity (HR 1.336, 95% CI 1.042–1.713), and high PM_2.5_ and high humidity (HR 1.664, 95% CI 1.294–2.140), again with the largest estimate for the high-high category. Among those in agricultural work, the excess risk was more modest, and only the high PM_2.5_ and high humidity category reached statistical significance (HR 1.347, 95% CI 1.085–1.671). Among those in non-agricultural work, none of the three categories showed statistically significant associations. Overall, these results suggest that participants without work were the most vulnerable employment subgroup in the population-based cohort.

In the single-center hospital-based study, the same two modifiers remained significant. Sex showed strong evidence of interaction (*P* for interaction <0.001). Among women, the effect estimates were consistently higher than in men, particularly for the combined high PM_2.5_–high humidity exposure. Specifically, high PM_2.5_ and high humidity was associated with markedly higher odds of stroke (OR 3.012, 95% CI 1.368–6.631), whereas high PM_2.5_ and low humidity (OR 1.938, 95% CI 0.920–4.082) and low PM_2.5_ and high humidity (OR 2.067, 95% CI 0.986–4.335) showed elevated but less precise estimates. Among men, the direction of association was similar, but only high PM_2.5_ and high humidity remained statistically significant (OR 2.203, 95% CI 1.061–4.577), while the other two exposure contrasts were positive but not statistically clear. These findings are consistent with greater vulnerability among women in the hospital-based sample.

Employment status also modified the exposure–stroke association in the hospital study (*P* for interaction = 0.003). Among participants with no work, the joint exposure categories were all associated with higher odds of stroke, including high PM_2.5_ and low humidity (OR 2.181, 95% CI 1.042–4.565), low PM_2.5_ and high humidity (OR 2.274, 95% CI 1.088–4.752), and high PM_2.5_ and high humidity (OR 3.284, 95% CI 1.575–6.845), with the strongest association again observed in the high-high category. By contrast, among those in agricultural work or non-agricultural work, effect estimates were directionally positive but less precise, and none of the joint exposure contrasts reached conventional statistical significance.

Consequently, the subgroup analyses suggest that the adverse association of joint PM_2.5_ and humidity exposure with stroke was not uniform across population strata. Across both datasets, women and participants without work tended to show the strongest associations, and in nearly all subgroup comparisons the high PM_2.5_ and high humidity category yielded the largest effect estimates relative to the low PM_2.5_ and low humidity reference group.

**Appendix 5. Sensitivity analysis excluding participants who developed stroke in the first two follow-up waves**

To reduce the possibility of reverse causation, we repeated the primary CHARLS analyses after excluding participants who developed incident stroke in the first two follow-up waves. A total of 642 early stroke events were excluded, leaving a restricted analytic sample of 16,555 participants, among whom 843 incident stroke events occurred during the remaining follow-up period. In the fully adjusted models, the associations of PM_2.5_, ambient relative humidity, and their joint exposure categories with incident stroke remained directionally consistent with the primary analysis and were only modestly attenuated in magnitude. High PM_2.5_ remained associated with a higher risk of incident stroke, as did high relative humidity. In the joint exposure analysis, the largest effect estimate continued to be observed in the high PM_2.5_ and high humidity category, followed by the high PM_2.5_ and low humidity category and the low PM_2.5_ and high humidity category. These findings suggest that the main results were not driven primarily by stroke events occurring shortly after baseline.

**Table A4.** Fully adjusted associations of PM_2.5_, ambient relative humidity, and their joint exposure categories with incident stroke in CHARLS after excluding participants who developed stroke in the first two follow-up waves

| **Exposure** | **HR (95% CI)** | **P-value** |
| --- | --- | --- |
| High PM_2.5_ vs. low PM_2.5_ | 1.161 (1.052–1.281) | 0.003 |
| High relative humidity vs. low relative humidity | 1.097 (1.001–1.203) | 0.048 |
| High PM_2.5_ and low humidity vs. low PM_2.5_ and low humidity | 1.214 (1.031–1.431) | 0.020 |
| Low PM_2.5_ and high humidity vs. low PM_2.5_ and low humidity | 1.176 (1.001–1.382) | 0.049 |
| High PM_2.5_ and high humidity vs. low PM_2.5_ and low humidity | 1.338 (1.138–1.573) | <0.001 |

**Note:** All estimates were derived from fully adjusted Cox proportional hazards models and were adjusted for age, sex, marital status, residence, smoking status, alcohol drinking status, income group, sleep duration group, number of living children, and employment status. The reference group in the joint exposure analysis was low PM_2.5_ and low humidity.

**Interpretation:** Compared with the primary analysis, exclusion of early incident stroke events resulted in slight attenuation of the effect estimates, but the overall pattern of association remained unchanged. In particular, the combined high PM_2.5_ and high humidity category continued to show the strongest association with incident stroke, supporting the robustness of the main findings and reducing concern that reverse causation substantially explained the observed results.

**Appendix 6. Sensitivity analysis using a 2-year lag annual average exposure window**

To examine whether the main findings were sensitive to the choice of exposure window, we repeated the primary analyses using a 2-year lag annual average exposure window instead of the primary 1-year lag window. The analytic samples were unchanged from the primary analyses, including 17,197 participants with 1,485 incident stroke events in CHARLS and 782 participants, including 148 stroke cases and 634 hospitalized non-stroke controls, in the hospital-based study. Overall, the results remained closely aligned with the main analysis. In both data sources, high PM_2.5_ and high relative humidity remained associated with higher stroke risk, and the largest effect estimates continued to be observed in the high PM_2.5_ and high humidity category. The magnitudes of association were only slightly attenuated compared with the primary models, indicating that the main conclusions were robust to use of a longer lagged exposure window.

**Table A5.** Associations of PM_2.5_, ambient relative humidity, and their joint exposure categories with stroke using a 2-year lag annual average exposure window

| **Exposure** | **CHARLS, HR (95% CI)** | **P value** | **Hospital-based study, OR (95% CI)** | **P value** |
| --- | --- | --- | --- | --- |
| High PM_2.5_ vs. low PM_2.5_ | 1.172 (1.069–1.284) | 0.001 | 1.781 (1.194–2.654) | 0.005 |
| High relative humidity vs. low relative humidity | 1.105 (1.013–1.206) | 0.024 | 1.864 (1.252–2.775) | 0.002 |
| High PM_2.5_ and low humidity vs. low PM_2.5_ and low humidity | 1.218 (1.042–1.425) | 0.013 | 1.652 (1.002–2.724) | 0.049 |
| Low PM_2.5_ and high humidity vs. low PM_2.5_ and low humidity | 1.181 (1.009–1.383) | 0.038 | 1.705 (1.031–2.820) | 0.038 |
| High PM_2.5_ and high humidity vs. low PM_2.5_ and low humidity | 1.346 (1.156–1.566) | <0.001 | 2.356 (1.331–4.171) | 0.003 |

**Note:** In CHARLS, estimates were derived from fully adjusted Cox proportional hazards models. In the hospital-based study, estimates were derived from fully adjusted multivariable logistic regression models. All models were adjusted for age, sex, marital status, residence, smoking status, alcohol drinking status, income group, sleep duration group, number of living children, and employment status. Exposure categories were defined using the database-specific median of the corresponding 2-year lag annual average exposure. In the joint exposure analysis, the reference group was low PM_2.5_ and low humidity.

**Interpretation:** Replacing the primary 1-year lag exposure window with a 2-year lag annual average window did not materially alter the overall pattern of association in either dataset. In particular, the combined high PM_2.5_ and high humidity category continued to show the strongest association with stroke, while the two intermediate joint exposure categories remained positively associated with stroke relative to the low PM_2.5_ and low humidity group. These findings suggest that the observed associations were not dependent on the specific lag structure used for exposure assignment and support the temporal robustness of the primary results.

**Appendix 7. Sensitivity analysis with additional adjustment for body mass index (BMI)**

To examine whether the primary findings were materially affected by adiposity, we repeated the fully adjusted models with additional adjustment for body mass index (BMI, kg/m^2^) in the subset of participants with valid BMI information. This sensitivity analysis was performed using complete-case data and included 14,908 participants with 1,302 incident stroke events in CHARLS and 721 participants, including 139 stroke cases and 582 hospitalized non-stroke controls, in the hospital-based study. Overall, the associations of PM_2.5_, ambient relative humidity, and their joint exposure categories with stroke remained very similar to those observed in the primary analysis. In both data sources, high PM_2.5_ and high humidity remained positively associated with stroke, and the combined high PM_2.5_ and high humidity category continued to show the largest effect estimate. These findings indicate that additional adjustment for BMI did not materially alter the overall pattern or interpretation of the main results.

**Table A6.** Associations of PM_2.5_, ambient relative humidity, and their joint exposure categories with stroke after additional adjustment for BMI

| **Exposure** | **CHARLS, HR (95% CI)** | **P value** | **Hospital-based study, OR (95% CI)** | **P value** |
| --- | --- | --- | --- | --- |
| High PM_2.5_ vs. low PM_2.5_ | 1.176 (1.068–1.295) | 0.001 | 1.829 (1.220–2.743) | 0.004 |
| High relative humidity vs. low relative humidity | 1.101 (1.004–1.207) | 0.041 | 1.891 (1.261–2.834) | 0.002 |
| High PM_2.5_ and low humidity vs. low PM_2.5_ and low humidity | 1.219 (1.038–1.432) | 0.015 | 1.671 (1.008–2.771) | 0.046 |
| Low PM_2.5_ and high humidity vs. low PM_2.5_ and low humidity | 1.181 (1.004–1.389) | 0.044 | 1.728 (1.039–2.875) | 0.035 |
| High PM_2.5_ and high humidity vs. low PM_2.5_ and low humidity | 1.352 (1.149–1.591) | <0.001 | 2.398 (1.348–4.267) | 0.003 |

**Note:** In CHARLS, estimates were derived from fully adjusted Cox proportional hazards models. In the hospital-based study, estimates were derived from fully adjusted multivariable logistic regression models. All models were adjusted for age, sex, marital status, residence, smoking status, alcohol drinking status, income group, sleep duration group, number of living children, employment status, and BMI. In the joint exposure analysis, the reference group was low PM_2.5_ and low humidity.

**Interpretation:** Additional adjustment for BMI resulted in only minimal changes in the effect estimates compared with the primary models. The persistence of the positive associations after BMI adjustment suggests that the observed relationships of PM_2.5_ and ambient humidity with stroke were not explained primarily by differences in adiposity. The retention of the largest effect estimate in the high PM_2.5_ and high humidity category further supports the robustness of the joint exposure findings.

**Appendix 8. Sensitivity analysis using a prospective discrete-time logistic regression model in CHARLS**

To evaluate whether the primary findings were sensitive to the choice of time-to-event modeling framework, we re-estimated the associations of PM_2.5_, ambient relative humidity, and their joint exposure categories with incident stroke in CHARLS using a prospective discrete-time logistic regression model. In this sensitivity analysis, the data were restructured into person-period format by follow-up wave, and time was explicitly accounted for in the model. Accordingly, incident stroke was modeled as occurring within a survey-wave interval rather than being assigned an assumed exact onset date. The person-period dataset included 61,084 person-wave observations and 1,485 incident stroke events. Overall, the effect estimates were closely comparable to those obtained from the primary Cox proportional hazards models. High PM_2.5_ and high relative humidity remained positively associated with incident stroke, and the largest effect estimate continued to be observed in the high PM_2.5_ and high humidity category. These findings suggest that the main results were robust to the choice of event-time analytical framework.

**Table A7.** Associations of PM_2.5_, ambient relative humidity, and their joint exposure categories with incident stroke in CHARLS using a prospective discrete-time logistic regression model

| **Exposure** | **OR (95% CI)** | **P value** |
| --- | --- | --- |
| High PM_2.5_ vs. low PM_2.5_ | 1.189 (1.087–1.302) | <0.001 |
| High relative humidity vs. low relative humidity | 1.118 (1.026–1.220) | 0.011 |
| High PM_2.5_ and low humidity vs. low PM_2.5_ and low humidity | 1.235 (1.060–1.439) | 0.007 |
| Low PM_2.5_ and high humidity vs. low PM_2.5_ and low humidity | 1.194 (1.022–1.394) | 0.025 |
| High PM_2.5_ and high humidity vs. low PM_2.5_ and low humidity | 1.371 (1.177–1.596) | <0.001 |

**Note:** Estimates were derived from a fully adjusted prospective discrete-time logistic regression model using person-period data structured by follow-up wave. Follow-up time was explicitly accounted for in the model. All estimates were adjusted for age, sex, marital status, residence, smoking status, alcohol drinking status, income group, sleep duration group, number of living children, and employment status. In the joint exposure analysis, the reference group was low PM_2.5_ and low humidity.

**Interpretation:** The discrete-time logistic regression model yielded effect estimates that were very similar in direction and magnitude to those from the primary Cox regression analyses. In particular, both PM_2.5_ and ambient relative humidity remained positively associated with incident stroke, and the joint exposure analysis again showed the highest estimate in the high PM_2.5_ and high humidity category. The close agreement between the discrete-time and Cox model results indicates that the main findings were not materially dependent on the specific time-to-event modeling strategy and supports the robustness of the primary conclusions.

**Appendix 9. Sensitivity analysis repeating the mediation analyses using categorical frailty status**

To assess whether the mediation findings were sensitive to the specification of the mediator, we repeated the mediation analyses using categorical frailty status instead of the continuous frailty index. Frailty status was defined dichotomously as non-frail versus frail using the conventional threshold of FI ≥ 0.25. Overall, the results were directionally consistent with the primary mediation analyses based on the continuous frailty index, although the indirect effects were slightly smaller and less precise. In both data sources, PM_2.5_ and ambient relative humidity continued to show significant natural indirect effects through frailty status, and the corresponding mediation proportions remained in a similar range to those observed in the main analyses. These findings supported the robustness of the model-based indirect associations in CHARLS and showed directionally consistent exploratory patterns in the hospital-based study. The hospital-based estimates should not be interpreted as establishing temporal or causal mediation.

**Table A8.** Counterfactual mediation analyses using categorical frailty status as the mediator

| **Exposure and outcome** | **Natural indirect effect, HR/OR (95% CI)** | **Natural direct effect, HR/OR (95% CI)** | **Total effect, HR/OR (95% CI)** | **Proportion mediated, % (95% CI)** |
| --- | --- | --- | --- | --- |
| **CHARLS** |  |  |  |  |
| PM_2.5_ and incident stroke | 1.027 (1.012–1.042) | 1.153 (1.053–1.263) | 1.184 (1.083–1.295) | 15.9 (8.4–24.2) |
| Ambient relative humidity and incident stroke | 1.022 (1.008–1.037) | 1.088 (0.999–1.186) | 1.112 (1.021–1.212) | 20.4 (10.9–30.7) |
| **Single-center hospital-based study** |  |  |  |  |
| PM_2.5_ and stroke | 1.088 (1.026–1.161) | 1.697 (1.135–2.538) | 1.846 (1.247–2.733) | 13.7 (5.2–23.6) |
| Ambient relative humidity and stroke | 1.103 (1.032–1.193) | 1.748 (1.169–2.614) | 1.928 (1.301–2.859) | 15.6 (6.3–25.9) |

**Note:** In this sensitivity analysis, frailty was modeled as a categorical mediator defined as frail (FI ≥ 0.25) versus non-frail (FI < 0.25). The mediation models were adjusted for age, sex, marital status, residence, smoking status, alcohol drinking status, income group, sleep duration group, number of living children, and employment status. Cox-based counterfactual mediation models were used for the CHARLS cohort, and logistic counterfactual mediation models were used for the hospital-based study. Confidence intervals were obtained using 5,000 bootstrap resamples.

**Interpretation:** When frailty was modeled categorically rather than continuously, the estimated mediated proportions were slightly attenuated, but the overall pattern remained directionally consistent with the primary analyses. The categorical-mediator results supported the robustness of the model-based indirect associations in CHARLS and showed similar exploratory patterns in the hospital-based study. The hospital-based estimates should not be interpreted as establishing temporal or causal mediation. The modest attenuation is also statistically plausible because dichotomization of the frailty index reduces information and may weaken sensitivity to gradations in vulnerability.

**Appendix 10. Sensitivity analysis with additional adjustment for the comorbidity index**

To examine whether the primary findings were materially altered after accounting for baseline chronic disease burden, we repeated the fully adjusted models with additional adjustment for a comorbidity index in both data sources. In the CHARLS cohort, the index was constructed from baseline chronic disease indicators, excluding baseline stroke because participants with prevalent stroke had already been removed from the analytic sample. In the single-center hospital-based study, the comorbidity index was derived from pre-existing chronic conditions documented in the admission record, past medical history section, discharge summary, and inpatient problem list, and was intended to reflect background multimorbidity burden rather than the study outcome itself. Overall, further adjustment for the comorbidity index resulted in only modest attenuation of the effect estimates. High PM_2.5_ and high ambient relative humidity remained positively associated with stroke in both datasets, and the combined high PM_2.5_ and high humidity category continued to show the largest effect estimate. These findings suggest that the main results were not explained primarily by baseline chronic disease burden.

**Table A9. Associations of PM_2.5_, ambient relative humidity, and their joint exposure categories with stroke after additional adjustment for the comorbidity index**

| **Exposure** | **CHARLS, HR (95% CI)** | **P value** | **Hospital-based study, OR (95% CI)** | **P value** |
| --- | --- | --- | --- | --- |
| High PM_2.5_ vs. low PM_2.5_ | 1.169 (1.064–1.283) | 0.001 | 1.768 (1.181–2.647) | 0.006 |
| High relative humidity vs. low relative humidity | 1.103 (1.009–1.206) | 0.031 | 1.846 (1.236–2.756) | 0.003 |
| High PM_2.5_ and low humidity vs. low PM_2.5_ and low humidity | 1.202 (1.031–1.401) | 0.019 | 1.624 (1.003–2.630) | 0.049 |
| Low PM_2.5_ and high humidity vs. low PM_2.5_ and low humidity | 1.173 (1.004–1.370) | 0.045 | 1.681 (1.029–2.748) | 0.038 |
| High PM_2.5_ and high humidity vs. low PM_2.5_ and low humidity | 1.329 (1.139–1.551) | <0.001 | 2.301 (1.294–4.092) | 0.004 |

**Note:** In CHARLS, estimates were derived from fully adjusted Cox proportional hazards models. In the hospital-based study, estimates were derived from fully adjusted multivariable logistic regression models. All models were additionally adjusted for the comorbidity index, along with age, sex, marital status, residence, smoking status, alcohol drinking status, income group, sleep duration group, number of living children, and employment status. In the joint exposure analysis, the reference group was low PM_2.5_ and low humidity. Detailed components and coding rules of the comorbidity index are provided in **Appendix 3**.

**Interpretation:** Additional adjustment for the comorbidity index led to slight attenuation of the associations in both datasets, but the overall pattern remained unchanged. In particular, the high PM_2.5_ and high humidity category continued to show the strongest association with stroke, and both PM_2.5_ and ambient relative humidity remained independently associated with less favorable stroke outcomes. These results support the robustness of the primary findings and suggest that the observed associations were not driven solely by differences in baseline multimorbidity burden.

**Appendix 11. Sensitivity analysis with additional adjustment for annual mean ambient temperature**

To examine whether the associations of PM_2.5_ and ambient relative humidity with stroke were materially influenced by the broader thermal environment, we repeated the fully adjusted models with additional adjustment for annual mean ambient temperature. Temperature was obtained from ERA5 and assigned according to the same residential location and one-year lagged exposure window used for relative humidity. Annual mean temperature was modeled as a continuous covariate. The analytic samples were unchanged from the primary analyses and included 17,197 participants with 1,485 incident stroke events in CHARLS and 782 participants, including 148 stroke cases and 634 hospitalized non-stroke controls, in the hospital-based study. Overall, temperature adjustment produced modest attenuation, particularly for the humidity-related estimates, while preserving the direction and ordering of the main associations. In both datasets, the high PM_2.5_ and high humidity category continued to show the largest effect estimate.

**Table A10. Associations of PM_2.5_, ambient relative humidity, and their joint exposure categories with stroke after additional adjustment for annual mean ambient temperature**

| **Exposure** | **CHARLS, HR (95% CI)** | **P value** | **Hospital-based study, OR (95% CI)** | **P value** |
| --- | --- | --- | --- | --- |
| High PM_2.5_ vs. low PM_2.5_ | 1.176 (1.073-1.289) | 0.001 | 1.792 (1.199-2.678) | 0.004 |
| High relative humidity vs. low relative humidity | 1.098 (1.005-1.199) | 0.038 | 1.741 (1.140-2.659) | 0.010 |
| High PM_2.5_ and low humidity vs. low PM_2.5_ and low humidity | 1.211 (1.039-1.411) | 0.014 | 1.637 (0.989-2.709) | 0.055 |
| Low PM_2.5_ and high humidity vs. low PM_2.5_ and low humidity | 1.161 (0.994-1.355) | 0.059 | 1.598 (0.954-2.676) | 0.075 |
| High PM_2.5_ and high humidity vs. low PM_2.5_ and low humidity | 1.327 (1.141-1.543) | <0.001 | 2.216 (1.238-3.966) | 0.007 |

**Note:** In CHARLS, estimates were derived from fully adjusted Cox proportional hazards models. In the hospital-based study, estimates were derived from fully adjusted multivariable logistic regression models. All models were adjusted for age, sex, marital status, residence, smoking status, alcohol drinking status, income group, sleep duration group, number of living children, employment status, and annual mean ambient temperature. Ambient temperature was assigned from ERA5 using the same residential location and one-year lagged exposure window as relative humidity and was modeled continuously. In the joint exposure analysis, the reference group was low PM_2.5_ and low humidity.

**Interpretation:** Additional adjustment for annual mean ambient temperature resulted in modest attenuation of the humidity-related estimates, as expected given the temperature dependence of relative humidity, but did not materially change the overall exposure-stroke pattern. High PM_2.5_ and high relative humidity remained independently associated with less favorable stroke outcomes in both data sources. Although the two intermediate joint exposure categories became less precise in the hospital-based study and the low PM_2.5_-high humidity category was borderline in CHARLS, the combined high PM_2.5_-high humidity category remained statistically significant and retained the largest effect estimate in both datasets. These findings indicate that the primary conclusions were not explained solely by differences in annual mean ambient temperature.

**Appendix 12. Spatial distribution, exposure characteristics, correlations, and spatial sensitivity analysis**

For supplementary exposure reporting, annual mean PM_2.5_ was assigned from the China High Air Pollutants (CHAP) surface at a spatial resolution of 1 km. Annual mean relative humidity and ambient temperature were derived from ERA5 data on a 0.25° × 0.25° latitude-longitude grid, corresponding to a nominal horizontal resolution of approximately 31 km. In CHARLS, exposure estimates were linked to geocoded residential-community centroids. In the hospital-based study, exposures were linked to the finest available residential geocode, with the centroid of the recorded county or district used when a complete address was unavailable. ERA5 values were assigned by bilinear interpolation from the surrounding grid cells. All three environmental variables were summarized over the calendar year immediately preceding baseline in CHARLS or the index date in the hospital-based study.

To provide additional information on the spatial distribution of the study sample, CHARLS participants were summarized across seven commonly used geographical regions. The regional distribution was based on province of residence and was used for descriptive presentation, whereas the spatial regression analysis used province-level fixed effects to provide finer control of broad geographic differences. East China accounted for the largest proportion of the analytic sample, followed by Southwest China and Central China. The environmental distributions showed the expected national gradients: annual mean relative humidity and ambient temperature were highest in South and East China and lowest in Northwest and North China, whereas annual mean PM_2.5_ concentrations were highest in North and Central China and lower in South and Southwest China.

**Table A11. Geographical distribution and annual environmental conditions in the CHARLS analytic sample**

| **Geographical region** | **Participants, n (%)** | **Annual mean PM_2.5_, mean (SD), μg/m³** | **Annual mean relative humidity, mean (SD), %** | **Annual mean ambient temperature, mean (SD), °C** |
| --- | --- | --- | --- | --- |
| Northeast China | 1,016 (5.9) | 51.0 (14.2) | 64.0 (4.2) | 6.4 (3.1) |
| North China | 1,842 (10.7) | 65.0 (17.4) | 56.5 (4.0) | 10.7 (3.2) |
| Northwest China | 1,446 (8.4) | 52.0 (14.7) | 47.5 (4.5) | 9.1 (4.7) |
| Central China | 2,651 (15.4) | 61.0 (15.5) | 69.5 (3.7) | 16.3 (2.4) |
| East China | 5,113 (29.7) | 55.8 (15.2) | 70.8 (3.5) | 16.9 (3.2) |
| Southwest China | 3,563 (20.7) | 45.0 (13.1) | 67.5 (4.2) | 14.1 (4.3) |
| South China | 1,566 (9.1) | 39.0 (11.7) | 77.0 (3.3) | 22.5 (2.0) |
| Total | 17,197 (100.0) | 53.2 (16.6) | 66.6 (8.6) | 14.8 (5.2) |

Geographical regions were defined according to province of residence using a conventional macroregional classification. Northeast China comprised Liaoning, Jilin, and Heilongjiang; North China comprised Beijing, Tianjin, Hebei, Shanxi, and Inner Mongolia; Northwest China comprised Shaanxi, Gansu, Qinghai, Ningxia, and Xinjiang; Central China comprised Henan, Hubei, and Hunan; East China comprised Shanghai, Jiangsu, Zhejiang, Anhui, Fujian, Jiangxi, and Shandong; Southwest China comprised Chongqing, Sichuan, Guizhou, Yunnan, and Tibet; and South China comprised Guangdong, Guangxi, and Hainan. Only provinces represented in CHARLS contributed observations. Values are presented as n (column proportion) or mean (SD). Percentages may not sum exactly to 100 because of rounding. Regional categories were used to describe spatial distribution and were not substituted for the province-level fixed effects used in the regression sensitivity analysis.

To address potential residual spatial confounding and correlation among participants residing in the same community, we repeated the primary CHARLS exposure analyses using robust sandwich variance estimates clustered at the residential-community level together with province-level fixed effects. The model therefore allowed standard errors to account for within-community correlation while controlling for broad province-level differences in climatic conditions, healthcare context, socioeconomic development, and other spatially patterned factors. The analysis included the same 17,197 participants distributed across 450 residential communities and retained the same individual-level covariates as the primary models.

**Table A12. Spatial sensitivity analysis of PM_2.5_, ambient relative humidity, and their joint exposure categories with incident stroke in CHARLS**

| **Exposure** | **HR (95% CI)** | **P value** |
| --- | --- | --- |
| High PM_2.5_ vs. low PM_2.5_ | 1.158 (1.042-1.287) | 0.007 |
| High relative humidity vs. low relative humidity | 1.095 (1.002-1.197) | 0.046 |
| High PM_2.5_ and low humidity vs. low PM_2.5_ and low humidity | 1.183 (0.992-1.411) | 0.062 |
| Low PM_2.5_ and high humidity vs. low PM_2.5_ and low humidity | 1.138 (0.952-1.360) | 0.155 |
| High PM_2.5_ and high humidity vs. low PM_2.5_ and low humidity | 1.298 (1.084-1.554) | 0.005 |

Estimates were derived from Cox proportional hazards models that included province-level fixed effects and robust sandwich variance estimates clustered at the residential-community level. All models were adjusted for age, sex, marital status, residence, smoking status, alcohol drinking status, income group, sleep duration group, number of living children, and employment status. The reference group in the joint exposure analysis was low PM_2.5_ and low humidity. The analysis included 17,197 participants from 450 residential communities across 28 provincial-level regions.

**Interpretation:** Province-level fixed effects and community-clustered robust variance estimates moderately attenuated the associations and widened the confidence intervals. The two intermediate joint exposure contrasts were no longer statistically significant, but high PM_2.5_, high relative humidity, and the high PM_2.5_-high humidity category remained significantly associated with incident stroke; the high-high category also retained the largest estimate. The principal conclusion was therefore robust to broad regional confounding and geographic clustering. This analysis was limited to CHARLS because community identifiers and sufficient province-level variation were available. Equivalent multilevel adjustment was not statistically appropriate in the hospital sample because suitable community cluster identifiers were unavailable and most participants resided in Fujian Province, providing insufficient between-province variation for stable province-level estimation.

The distributions of the three continuous environmental exposures were further summarized using dataset-specific medians and observed ranges. The substantially broader ranges in CHARLS reflected its national geographic coverage, whereas the narrower hospital-based ranges were consistent with the concentration of participants in Fujian Province and nearby areas.

**Table A13. Median and observed range of annual environmental exposures in the two study datasets**

| **Data source** | **Exposure** | **Median** | **Observed range** |
| --- | --- | --- | --- |
| CHARLS | Annual mean PM_2.5_, μg/m^3^ | 52.1 | 18.6-94.2 |
| CHARLS | Annual mean relative humidity, % | 67.0 | 46.1-85.7 |
| CHARLS | Annual mean ambient temperature, °C | 15.2 | -6.8 to 27.1 |
| Hospital-based study | Annual mean PM_2.5_, μg/m^3^ | 28.8 | 10.5-52.4 |
| Hospital-based study | Annual mean relative humidity, % | 74.2 | 53.8-91.6 |
| Hospital-based study | Annual mean ambient temperature, °C | 20.7 | 14.6-26.1 |

Exposures were assigned according to residential location for the calendar year immediately preceding baseline in CHARLS or the index date in the hospital-based study. The observed range represents the minimum and maximum participant-level assigned values in each analytic dataset.

Pairwise correlations among continuous annual mean PM_2.5_, relative humidity, and ambient temperature were assessed separately in the two datasets using Spearman rank correlation coefficients.

**Table A14. Pairwise Spearman correlations among annual mean PM_2.5_, relative humidity, and ambient temperature in the two study datasets**

| **Data source** | **Exposure pair** | **Spearman ρ** | **P value** |
| --- | --- | --- | --- |
| CHARLS | PM_2.5_ and relative humidity | -0.214 | <0.001 |
| CHARLS | PM_2.5_ and ambient temperature | -0.251 | <0.001 |
| CHARLS | Relative humidity and ambient temperature | 0.708 | <0.001 |
| Hospital-based study | PM_2.5_ and relative humidity | 0.438 | <0.001 |
| Hospital-based study | PM_2.5_ and ambient temperature | 0.287 | <0.001 |
| Hospital-based study | Relative humidity and ambient temperature | 0.603 | <0.001 |

Correlations were calculated using participant-level continuous exposure values assigned for the same one-year lagged annual average window. Negative values indicate inverse rank correlations and positive values indicate positive rank correlations. The strongest correlation was observed between relative humidity and ambient temperature, consistent with the temperature dependence of relative humidity; however, no pairwise correlation approached unity.

**Appendix 13. Detailed results and interpretation of the sensitivity analyses**

Eight sensitivity analyses were conducted to evaluate the robustness of the primary findings. First, in the CHARLS cohort, after excluding participants who developed stroke in the first two follow-up waves (excluded early events = 642; restricted n = 16,555; late incident strokes = 843), the associations remained directionally consistent and materially unchanged. In fully adjusted models, high PM_2.5_ remained associated with higher incident stroke risk (HR = 1.161, 95% CI 1.052–1.281), as did high relative humidity (HR = 1.097, 95% CI 1.001–1.203). In the joint exposure analysis, compared with low PM_2.5_ and low humidity, the HRs were 1.214 (95% CI 1.031–1.431) for high PM_2.5_ and low humidity, 1.176 (95% CI 1.001–1.382) for low PM_2.5_ and high humidity, and 1.338 (95% CI 1.138–1.573) for high PM_2.5_ and high humidity **(Appendix 5)**.

Second, when the exposure window was redefined using a 2-year lag annual average instead of the primary 1-year lag, effect estimates in both data sources remained closely aligned with the main analysis. In CHARLS, high PM_2.5_ was associated with an HR of 1.172 (95% CI 1.069–1.284), high humidity with an HR of 1.105 (95% CI 1.013–1.206), and the high PM_2.5_ and high humidity category with an HR of 1.346 (95% CI 1.156–1.566). In the hospital-based study, the corresponding estimates were OR = 1.781 (95% CI 1.194–2.654), OR = 1.864 (95% CI 1.252–2.775), and OR = 2.356 (95% CI 1.331–4.171), respectively **(Appendix 6)**.

Third, additional adjustment for BMI in the subset with valid BMI information produced results very similar to the primary models. In CHARLS (n = 14,908; incident strokes = 1,302), high PM_2.5_ remained associated with incident stroke (HR = 1.176, 95% CI 1.068–1.295), high humidity remained associated with incident stroke (HR = 1.101, 95% CI 1.004–1.207), and the high PM_2.5_ and high humidity group retained the largest effect estimate (HR = 1.352, 95% CI 1.149–1.591). In the hospital-based subset with BMI data (n = 721; stroke cases = 139), the corresponding estimates were OR = 1.829 (95% CI 1.220–2.743), OR = 1.891 (95% CI 1.261–2.834), and OR = 2.398 (95% CI 1.348–4.267) **(Appendix 7)**.

Fourth, in the CHARLS cohort, re-estimating the associations using a prospective discrete-time logistic regression model yielded effect estimates that closely matched those from the primary Cox models. In the person-period dataset (61,084 person-wave observations; 1,485 incident stroke events), high PM_2.5_ was associated with an OR of 1.189 (95% CI 1.087–1.302), high humidity with an OR of 1.118 (95% CI 1.026–1.220), and the high PM_2.5_ and high humidity category with an OR of 1.371 (95% CI 1.177–1.596) **(Appendix 8)**.

Fifth, repeating the mediation analyses using categorical frailty status instead of the continuous frailty index produced slightly less precise but directionally consistent results. In CHARLS, the mediation proportions were 15.9% (95% CI 8.4%–24.2%) for PM_2.5_ and 20.4% (95% CI 10.9%–30.7%) for humidity. In the hospital-based study, the corresponding mediation proportions were 13.7% (95% CI 5.2%–23.6%) and 15.6% (95% CI 6.3%–25.9%), respectively **(Appendix 9)**.

Sixth, after additional adjustment for the comorbidity index in both data sources, the main findings were modestly attenuated but remained statistically significant. In CHARLS, the HRs were 1.169 (95% CI 1.064–1.283) for high PM_2.5_, 1.103 (95% CI 1.009–1.206) for high humidity, and 1.329 (95% CI 1.139–1.551) for the high PM_2.5_ and high humidity category. In the hospital-based study, the corresponding ORs were 1.768 (95% CI 1.181–2.647), 1.846 (95% CI 1.236–2.756), and 2.301 (95% CI 1.294–4.092), respectively **(Appendix 10)**.

Seventh, to evaluate potential confounding by the broader thermal environment, the fully adjusted models were additionally adjusted for annual mean ambient temperature assigned from ERA5 using the same residential location and 1-year lagged exposure window as relative humidity. Temperature adjustment resulted in modest attenuation, particularly for the humidity-related estimates, but did not materially alter the overall pattern. In CHARLS, the HRs were 1.176 (95% CI 1.073–1.289) for high PM_2.5_, 1.098 (95% CI 1.005–1.199) for high relative humidity, and 1.327 (95% CI 1.141–1.543) for the high PM_2.5_ and high humidity category. In the hospital-based study, the corresponding ORs were 1.792 (95% CI 1.199–2.678), 1.741 (95% CI 1.140–2.659), and 2.216 (95% CI 1.238–3.966), respectively. Although the two intermediate joint exposure categories became less precise after temperature adjustment, the combined high PM_2.5_ and high humidity category remained statistically significant and retained the largest effect estimate in both datasets **(Appendix 11)**.

Finally, potential broad regional differences and correlation among participants residing within the same community were evaluated in CHARLS by including province-level fixed effects and using robust sandwich variance estimates clustered at the residential-community level. This analysis included 17,197 participants distributed across 450 residential communities and 28 provincial-level regions. The resulting HRs were 1.158 (95% CI 1.042–1.287) for high PM_2.5_, 1.095 (95% CI 1.002–1.197) for high relative humidity, and 1.298 (95% CI 1.084–1.554) for the high PM_2.5_ and high humidity category. The two intermediate joint exposure contrasts were attenuated and were no longer statistically significant, whereas the high PM_2.5_ and high humidity category remained statistically significant and retained the largest effect estimate. Thus, the principal high-exposure pattern was not materially altered after controlling for broad province-level differences and accounting for within-community correlation **(Appendix 12)**. This spatial analysis was limited to CHARLS because suitable residential-community identifiers and sufficient province-level variation were available in that dataset, whereas most hospital-based participants resided in Fujian Province and consistent community-level cluster identifiers were unavailable.

Collectively, excluding early events, extending the lag window, additional adjustment for BMI and baseline multimorbidity, using an alternative discrete-time modeling framework, replacing the continuous mediator with categorical frailty, controlling for annual mean ambient temperature, and accounting for broad province-level differences and within-community correlation did not materially alter the principal conclusions. Although some intermediate joint exposure estimates were attenuated in the temperature- and spatially adjusted analyses, the high PM_2.5_ and high humidity category consistently retained the largest effect estimate across the sensitivity analyses.
